# Foxm1 regulates cardiomyocyte proliferation in adult zebrafish after cardiac injury

**DOI:** 10.1242/dev.201163

**Published:** 2023-03-14

**Authors:** Daniel A. Zuppo, Maria A. Missinato, Lucas Santana-Santos, Guang Li, Panayiotis V. Benos, Michael Tsang

**Affiliations:** ^1^ Department of Developmental Biology, University of Pittsburgh, School of Medicine, Pittsburgh, PA 15213, USA; ^2^ Avidity Biosciences, 10578 Science Center Dr. Suite 125, San Diego, CA 92121, USA; ^3^ Department of Computational and Systems Biology, University of Pittsburgh, School of Medicine, Pittsburgh, PA 15213, USA

**Keywords:** Heart regeneration, Foxm1, Cardiomyocyte proliferation, Cenpf, Binucleation, Zebrafish

## Abstract

The regenerative capacity of the mammalian heart is poor, with one potential reason being that adult cardiomyocytes cannot proliferate at sufficient levels to replace lost tissue. During development and neonatal stages, cardiomyocytes can successfully divide under injury conditions; however, as these cells mature their ability to proliferate is lost. Therefore, understanding the regulatory programs that can induce post-mitotic cardiomyocytes into a proliferative state is essential to enhance cardiac regeneration. Here, we report that the forkhead transcription factor Foxm1 is required for cardiomyocyte proliferation after injury through transcriptional regulation of cell cycle genes. Transcriptomic analysis of injured zebrafish hearts revealed that *foxm1* expression is increased in border zone cardiomyocytes. Decreased cardiomyocyte proliferation and expression of cell cycle genes in *foxm1* mutant hearts was observed, suggesting it is required for cell cycle checkpoints. Subsequent analysis of a candidate Foxm1 target gene, *cenpf*, revealed that this microtubule and kinetochore binding protein is also required for cardiac regeneration. Moreover, *cenpf* mutants show increased cardiomyocyte binucleation. Thus, *foxm1* and *cenpf* are required for cardiomyocytes to complete mitosis during zebrafish cardiac regeneration.

## Teaser

**Summary:** This study highlights the importance of Foxm1, a transcription factor that is activated in cardiac muscle cells to regulate cell cycle gene expression and cell division in regeneration.

## INTRODUCTION

Cardiac regeneration is a phenomenon where multiple cell types respond after injury to replace damaged heart tissue. Urodele amphibians (Becker et al., 1974; Oberpriller and Oberpriller, 1974), zebrafish (Chablais et al., 2011; Gonzalez-Rosa et al., 2011; Poss et al., 2002; Raya et al., 2003) and neonatal mice (Porrello et al., 2011) possess this regenerative capacity, whereas others, such as medaka (Ito et al., 2014) and adult mammals (Rumyantsev, 1977), lack this ability. A major difference between regenerative and non-regenerative hearts is whether cardiomyocytes divide after injury to replenish the damaged tissue. Although some species retain this ability throughout their lifespan (Becker et al., 1974; Chablais et al., 2011; Gonzalez-Rosa et al., 2011; Oberpriller and Oberpriller, 1974; Poss et al., 2002; Raya et al., 2003), others possess a limited proliferative window that is lost as cardiomyocytes mature. In mice, neonatal cardiomyocytes can robustly divide after injury, but division rapidly decreases after post-natal day (P) 7 and fibrotic tissue persists at the injury site (Porrello et al., 2011). Understanding how this proliferative window is determined would allow us to stimulate cardiomyocyte proliferation in non-regenerative, injurysettings.

Embryonic cardiomyocytes activate multiple pathways that promote maturation within these cells. These processes include changes in sarcomere assembly, metabolism and cell cycle activity. Loosely organized myofibrils are produced in embryonic cardiomyocytes at the beginning of sarcomere assembly, but a large expansion in myofibril number occurs during cell maturation coupled with increased organization of the sarcomere into banded, contractile structures of the Z-line and M-line (Guo and Pu, 2020; Padula et al., 2021). In addition to this, mouse embryonic cardiomyocytes express genes encoding sarcomere components, such as *Myh7*, *Myl7*, and *Tnni1*, whereas adult cells predominantly express *Myh6*, *Myl2* and *Tnni3* after maturation (Guo and Pu, 2020; Padula et al., 2021). With increased contractile organization, adult cardiomyocytes also require higher levels of ATP to sustain cardiac function. Mature cardiomyocytes use fatty acid oxidation to generate substrates required for ATP generation via oxidative phosphorylation (Sartiani et al., 2007). These cells have increased mitochondria number, size, and more developed cristae that support the high levels of ATP required for contraction in adult cardiomyocytes (Papanicolaou et al., 2012). In contrast, embryonic cardiomyocytes are reliant on glycolysis as their primary energy source, which is shown through increased expression of the glycolysis initiation gene hexokinase 1 (*Hk1*) (Fritz et al., 1999) and the presence of immature mitochondria (Dai et al., 2017). Cell cycle activity is another hallmark that indicates the maturation status of cardiomyocytes as they switch from a proliferative state in embryonic cells and exit the cell cycle as they mature. During mouse development, cardiomyocyte proliferation peaks at embryonic day (E) 12 and P4-6. These cells have increased mRNA and protein levels of cyclins and cyclin-dependent kinases (CDKs) required for cell cycle progression (Kang et al., 1997; Soonpaa et al., 1996; Walsh et al., 2010). However, the expression of cyclin-dependent kinase inhibitors (CKIs) increases by P3-P6 and remains elevated in cardiomyocytes, preventing cell cycle progression (Ikenishi et al., 2012; Kang et al., 1997; Soonpaa et al., 1996; Tane et al., 2014). Concurrent with the reduction in cycling cardiomyocytes, the ploidy type begins to switch from mononucleated diploid to either mononucleated or binucleated polyploid cells (Bergmann et al., 2015; Mollova et al., 2013; Soonpaa et al., 1996). Taken together, this suggests that multiple regulatory programs coordinate to initiate cardiomyocyte maturation and attenuate the proliferation of these cells. Once cardiomyocytes mature in mammals, it becomes increasingly difficult to drive them into a regenerative state and this is one of the reasons why fibrotic tissue remains after cardiac injury.

Unlike mammals, adult zebrafish cardiomyocytes can dedifferentiate from a mature to an embryonic state and exhibit robust proliferation under different injury conditions (Gonzalez-Rosa et al., 2011; Poss et al., 2002; Raya et al., 2003; Wang et al., 2011). This makes them an ideal model to study which genes regulate the proliferative switch in these cells. In the cardiac resection model, 20% of the ventricle is surgically removed and a clot forms at the site of injury (Poss et al., 2002). Cardiomyocytes respond to induced stimuli at 3 days post-amputation (dpa) and begin to dedifferentiate and divide. By 7 dpa, cardiomyocyte proliferation peaks, and it decreases by 14 dpa. Using this model, studies have shown that cardiomyocyte proliferation can be activated via multiple factors (Gemberling et al., 2015; Han et al., 2019; Lien et al., 2006; Wu et al., 2016). However, many of these genes encode secreted ligands involved in cell non-autonomous activation of the cell cycle and the identification of downstream transcription factors required for cardiomyocyte proliferation is not well characterized.

In this study, RNA-sequencing (RNA-seq) was performed on uninjured and resected hearts to identify genes involved in mitotic regulation. *Forkhead box M1* (*foxm1*), a transcription factor, was significantly increased at 3 dpa and was deemed a candidate for injury-induced cardiomyocyte proliferation based on its known role in driving proliferation in cancer (Kalin et al., 2006; Kim et al., 2006; Madureira et al., 2006; Wang et al., 2008). Indeed, we show that *foxm1* is expressed in a subset of cardiomyocytes within the injury zone and that *foxm1* mutant hearts displayed significantly decreased cardiomyocyte proliferation. Transcriptome analysis of *foxm1* mutants revealed decreased expression of activator protein-1 (AP-1), glucose metabolism and G_2_/M phase cell cycle genes, indicating impaired cardiomyocyte proliferation. Of these G_2_/M genes, *centromere protein F* (*cenpf*), a canonical target of Foxm1, is required for the completion of chromatin segregation, and its depletion induces mitotic arrest in mammalian cells (Holt et al., 2005; Laoukili et al., 2005; Liao et al., 1995; Rattner et al., 1993). *cenpf* mutant hearts accumulate more binucleated cells, revealing a failure to complete mitosis. This study demonstrates the importance of Foxm1 as a regulator of cell cycle progression, but also reveals a role for Cenpf in cardiomyocyte mitosis during heart regeneration.

## RESULTS

### Identification of mitotic genes involved in cardiac regeneration

**Fig. 1.**
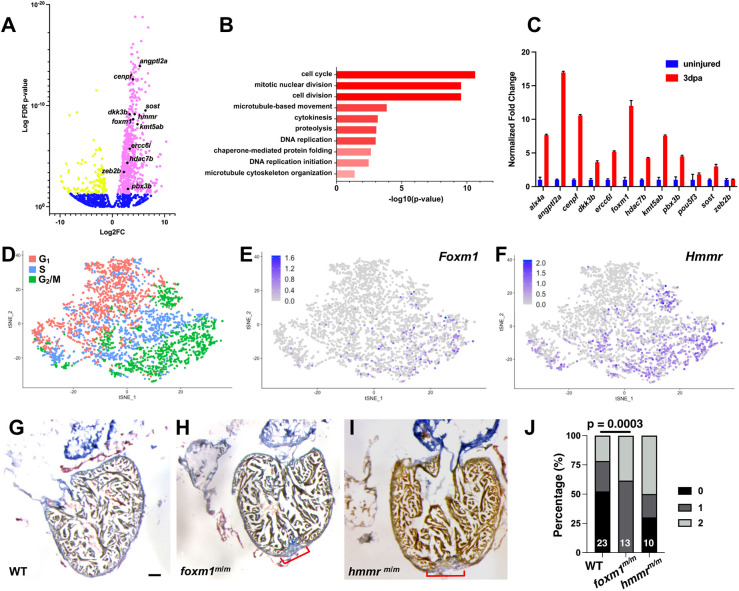
**Identification of mitotic genes involved in cardiac regeneration.** (A) Representative volcano plot of RNA-seq from uninjured versus 3 dpa adult ventricles. Magenta denotes genes with increased transcripts at 3 dpa, yellow represents decreased transcripts and blue represents genes with no significant change. (B) Genes with increased transcription were analyzed with FAC (DAVID) and biological processes were ranked by *z*-score. (C) qPCR validation was performed on candidate genes and the representative image shown depicts fold change+s.d. (D) scRNA-seq showed E10.5 embryonic left ventricular cardiomyocytes sorted by cell cycle phase. (E,F) *Foxm1* (E) and *Hmmr* (F) were present in G2 and M phase cardiomyocytes. (G-I) Representative images from WT 30 dpa (G; *n*=22), *foxm1^m/m^* 30 dpa (H; *n*=13) and *hmmr^m/m^* 30 dpa (I; *n*=7) hearts stained with AFOG. AFOG staining labels fibrin (red), collagen (blue) and muscle (orange-brown). Red brackets indicate presence of fibrotic tissue. (J) Scar area was qualitatively scored: 0, no visible scar; 1, small amount of fibrosis with some collagen and fibrin stain; 2, medium to large amount of fibrosis with collagen and fibrin stain. Increased fibrotic area was noted in *foxm1* mutant hearts compared with WT controls. Statistical significance was calculated using Fisher's exact test. Scale bars: 50 μm.

We previously determined that Dusp6, an ERK phosphatase, limits heart regeneration in zebrafish (Missinato et al., 2018). Heart regeneration in *dusp6* mutants was accelerated due to increased cardiomyocyte proliferation and neovascularization. To investigate transcriptome changes during the early stages of cardiac regeneration, we analyzed differential gene expression (DGE) in adult hearts collected from uninjured or injured WT and *dusp6* mutant zebrafish. Ventricular amputated hearts were extracted at 3, 7 and 20 dpa, and total RNA was isolated for RNA-seq. For analysis, wild-type (WT) and *dusp6* mutant data were combined based on their injury status as these were single bulk RNA-seq experiments. DGE analysis revealed that 1149 genes were significantly increased, whereas 153 genes were decreased, at 3 dpa (Fig. 1A; Table S1). Genes with increased expression at 3 dpa included *ect2*, *plk1* and *hmmr* (Gonzalez-Rosa et al., 2018; Jopling et al., 2010; Missinato et al., 2015). These genes are known to play crucial roles in zebrafish cardiac regeneration and detecting these transcripts demonstrated that these injured hearts were in a proliferative state (Table S1). Consistently, using functional annotation clustering (FAC), DAVID gene ontology showed an enrichment of genes involved in specific biological processes including cell cycle, cell division and DNA-binding genes (Fig. 1B; Table S2). To validate the RNA-seq data, qPCR was performed using uninjured and 3 dpa hearts to confirm that candidate genes were increased upon injury (Fig. 1C). qPCR revealed increased expression of DNA-binding factors (*alx4a*, *foxm1*, *pbx3b*) and chromatin modifiers (*kmt5ab* and *hdac7b*) but not stemness factors (*pou5f3* and *zeb2b*) (Fig. 1C).

*hmmr* and *foxm1* were selected as candidates for further analysis, as their expression was also increased at 7 dpa but was comparable with uninjured adult hearts at 20 dpa (Fig. S1; Table S1). These genes are known to be active during mammalian heart development (Bolte et al., 2011; Camenisch et al., 2000; Ramakrishna et al., 2007) and mitosis (Dunsch et al., 2012; Korver et al., 1997, 1998). *Hmmr* (hyaluronan mediated motility receptor) is implicated in cell proliferation via Erk1/2 phosphorylation (Hatano et al., 2012) and spindle fiber orientation (Dunsch et al., 2012). It is expressed in the cardiac jelly during heart development (Camenisch et al., 2000), and *hmmr* activates zebrafish epicardial cells during cardiac regeneration (Missinato et al., 2015). *Foxm1* is a transcription factor involved in cell cycle progression that promotes the expression of downstream genes necessary for mitotic completion (Korver et al., 1997, 1998; Laoukili et al., 2005). Expression of *Foxm1* is detected in the compact myocardium, trabeculated myocardium and endothelial cells during murine heart development (Bolte et al., 2011; Ramakrishna et al., 2007) and *Foxm1* is required for cardiomyocyte proliferation (Bolte et al., 2011; Ramakrishna et al., 2007; Sengupta et al., 2013) in the embryonic mouse heart. Our analysis of *foxm1* and *hmmr* transcript in regenerating zebrafish hearts shows increased expression during stages where cardiomyocyte proliferation is active (Fig. S1) We confirmed that *Foxm1* and *Hmmr* are expressed in cycling cardiomyocytes from single cell RNA-seq (scRNA-seq) data of E10.5 embryonic mouse hearts (Fig. 1D-F). *Foxm1* and *Hmmr* expression was enriched in G_2_/M phase cardiomyocytes in the mouse heart, suggesting that they may play an important role for cycling cells during regeneration.

To determine whether these genes were required for cardiac regeneration, *foxm1^sa10708/10708^* (referred to as *foxm1^m/m^*) and *hmmr^sa12528/12528^* (referred to as *hmmr^m/m^*) mutant lines from the Sanger zebrafish mutagenesis project were studied. In both cases, homozygous mutants were viable as adults and appeared normal. It was surprising that loss of *foxm1* does not result in an embryonic phenotype as has been reported in mice (Bolte et al., 2011; Sengupta et al., 2013). We did note that adult male *foxm1* homozygous mutants are infertile and have never been able to successfully fertilize eggs. The *foxm1* ENU-induced mutation from the Sanger Consortium is predicted to be within the forkhead DNA binding domain. Thus, we believe that the mutation is damaging, and it is not likely a splice variant can compensate for the forkhead DNA binding function. One reason for the lack of embryonic phenotype is that related Fox genes may compensate for the loss of *foxm1* during development. We performed ventricle resection and collected hearts at 30 dpa to determine whether cardiac regeneration was impaired (Fig. 1G-J). Semi-qualitative analysis of the fibrotic area was performed to indicate the increased severity of the damage as shown in previous studies (Bise et al., 2020; She et al., 2020; Sun et al., 2022; Xia et al., 2022). Acid fuchsin orange G (AFOG) staining revealed fibrotic tissue in *foxm1^m/m^* (Fig. 1H; Fig. S2) and *hmmr^m/m^* (Fig. 1I; Fig. S2) hearts at 30 dpa compared with WT controls (Fig. 1G; Fig. S2), and a significant difference was observed in *foxm1^m/m^* 30 dpa hearts (Fig. 1J). This suggests that *foxm1* and *hmmr* are important for the progression of cardiac regeneration and their loss inhibits crucial processes required for fibrotic resolution.

### *foxm1* is expressed in border zone cardiomyocytes and is required for proliferation after ventricular resection

**Fig. 2.**
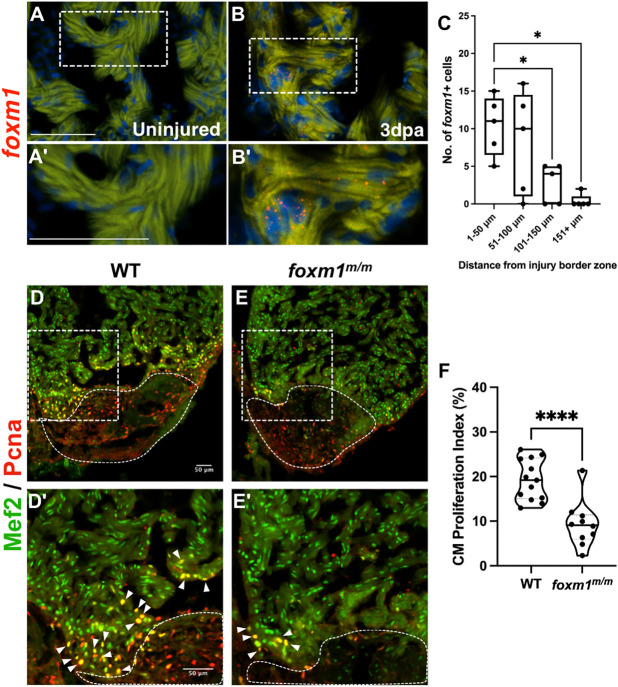
**Loss of *foxm1* significantly limits cardiomyocyte proliferation after ventricular resection.** (A-B′) Fluorescence *in situ* hybridization revealed *foxm1* (red) is not expressed in the uninjured myocardium (A,A′) but is detected in border zone cardiomyocytes after injury (B,B′). The myocardium is marked by 488 nm as background fluorescence. (C) *foxm1*^+^ cardiomyocytes were localized within 100 µm from the injury and expression decreased distal to the wound. Box plots show minimum to maximum. The whiskers show the minimum and up to the maximum value, with the middle line representing the median. Each point represents *foxm1*^+^ cardiomyocytes from each heart with respect to distance from the border zone. Each point is representative of an individual heart (*n*=5). (D-E′) WT (D,D′; *n*=13) and *foxm1^m/m^* (E,E′; *n*=10) hearts at 7 dpa stained for the cardiomyocyte nuclei marker Mef2 (green) and proliferation marker Pcna (red). White dotted lines represent the injury border. Arrowheads indicate proliferating cardiomyocytes. (F) Cardiomyocyte proliferation index is significantly decreased in *foxm1^m/m^* hearts. Each point on the truncated violin plot represents an individual heart and these data represent three biological replicates. The middle line represents the median. Statistical significance was calculated using one-way ANOVA multiple comparison test. *P<0.05, ****P<0.0001. A′,B′,D′,E′ show magnified images of boxed areas in A,B,D,E, respectively. Scale bars: 50 μm.

As Hmmr has previously been studied in mice and zebrafish (Camenisch et al., 2000; Missinato et al., 2015), we focused on *foxm1*, as it is not known which cells express this transcription factor during adult zebrafish cardiac regeneration. Fluorescence *in situ* hybridization showed that *foxm1* mRNA was expressed in myocardium after injury (Fig. 2A-B′). *foxm1*^+^ cardiomyocyte distance from the wound border was quantified and *foxm1*^+^ cells were within 100 µm from the injury plane at 3 dpa (Fig. 2C). The proximity of these *foxm1*^+^ cardiomyocytes to the clot suggests that they localized in the border zone myocardium, a tissue area with active proliferation following ventricular resection. As further confirmation, *Tg(myl7*:*EGFP)* and *Tg(wt1b*:*EGFP)* hearts, which label cardiomyocytes and epicardial cells, respectively, were used to confirm *foxm1* expression in cardiomyocytes (Fig. S3). In contrast, *wt1b*:*EGFP*^+^ epicardial cells did not express *foxm1* (Fig. S3). This does not exclude the possibility that *foxm1* transcript is not expressed in other cells residing in the myocardium, such as endocardial cells (Bolte et al., 2011), but these data suggest that *foxm1* expression is predominantly localized to border zone cardiomyocytes after cardiac injury.

We next explored whether *foxm1* was required for the proliferation of these cells after ventricular resection. Using WT (Fig. 2D,D′) and *foxm1^m/m^* (Fig. 2E,E′) 7 dpa hearts, we measured the proliferation index and observed a significant decrease in Mef2^+^/Pcna^+^ cardiomyocytes in *foxm1* mutants (Fig. 2F). Together, these findings show that *foxm1* is required for cardiomyocyte proliferation after cardiac injury. Although *foxm1* is also expressed in other cell types during development, including the endocardium, we have not ruled out proliferation deficiency in this population.

### *foxm1* is epistatic to *dusp6* and the Ras/MAPK pathway

**Fig. 3.**
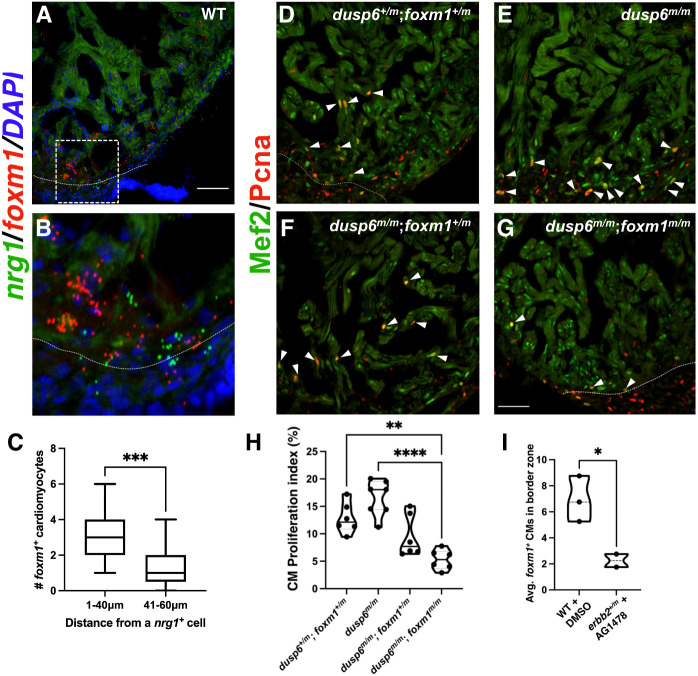
***foxm1*^+^ cardiomyocytes are localized near *nrg1*^+^ cells and are epistatic to the Mapk regulator, *dusp6*.** (A,B) *In situ* hybridization showing *nrg1* (green) and *foxm1* (red) in WT 7 dpa hearts. (C) *foxm1*^+^ cardiomyocytes were proximal to *nrg1*^+^ non-myocytes. A total of 21 *nrg1*^+^ cells were scored in WT 7 dpa hearts (*n*=5). Box plots show minimum to maximum. The whiskers show the minimum and up to the maximum value, with the middle line representing the median. (D-G) *dusp6^m/^*^+^;*foxm1^m/^*^+^ fish were bred to generate double mutants to assess their epistatic relationship. *dusp6^m/m^* hearts (E; *n*=7), *dusp6^m/^*^+^;*foxm1^m/^*^+^ (D; *n*=6), *dusp6^m/m^*;*foxm1^m/^*^+^ hearts (F; *n*=6) and *dusp6^m/m^*;*foxm1^m/m^* hearts (G; *n*=6) were stained with Mef2 (green) and Pcna (red). White dotted lines represent the injury border. White arrowheads indicate representative Pcna^+^/Mef2^+^ cardiomyocytes. (H) Graph showing CM proliferation index. Each point on the truncated violin plot represents an individual heart and these data represent three biological replicates. The middle line represents the median. (I) *In situ* hybridization for *foxm1* was performed in WT (*n*=3) vehicle treated with 50% DMSO and *erbb2^+/m^* (*n*=2) hearts treated with AG1478. The number of *foxm1*^+^ cardiomyocytes was quantified in control and *erbb2^+/m^*-treated hearts. Each point on the truncated violin plot represents an individual heart and is averaged from four sections. The middle line represents the median. Statistical significance was calculated using two-tailed, unpaired Student's *t*-test and one-way ANOVA multiple comparison test. ***P=0.0006 (two-tailed, unpaired Student's *t*-test, C); **P=0.0022, ****P<0.0001 (one-way ANOVA, H); *P=0.0416 (two-tailed, unpaired Student's *t*-test, I). Scale bars: 50 μm.

We next sought to identify potential upstream pathways that regulate *foxm1* expression and activity. The EGF family ligand Neuregulin 1 (Nrg1) is expressed in the endocardium and coronary artery microvasculature in adult, mammalian hearts (Liu et al., 2006). Nrg1 can induce cardiomyocyte division post-injury following injection of recombinant NRG1 or constitutive activation of its cognate receptor Erbb2 (Bersell et al., 2009; D'Uva et al., 2015). In adult zebrafish, *nrg1* is expressed in epicardial cells from the perivascular compartment as well as regulatory T cells, and both cell types can stimulate cardiomyocyte proliferation after ventricular resection (Gemberling et al., 2015; Hui et al., 2017). Therefore, we hypothesized that *foxm1* expression in cardiomyocytes is localized near *nrg1*^+^ cells that infiltrate the myocardium post-injury. Double *in situ* hybridization revealed *nrg1*^+^ cells near the site of injury and were often flanked by multiple *foxm1*^+^ cardiomyocytes (Fig. 3A,B). The number of *foxm1*^+^ cardiomyocytes near *nrg1^+^* non-myocytes was counted and a higher incidence of expression of these two genes in proximity was observed (Fig. 3C). As Nrg1 is known to activate the Ras/Mapk pathway and FOXM1 nuclear localization is regulated by ERK phosphorylation (Ma et al., 2005), we explored the genetic interaction between *dusp6* and *foxm1*. Given that *dusp6^m/m^* hearts show increased cardiomyocyte proliferation after injury (Missinato et al., 2018), we hypothesized that the *foxm1* mutation would be sufficient to block the pro-regenerative phenotype present in *dusp6* mutants. Double *dusp6:foxm1* mutant hearts showed a significant decrease in cardiomyocyte proliferation when compared with *dusp6^m/m^* hearts at 7 dpa (Fig. 3D-H). This demonstrates that the loss of *foxm1* is sufficient to block the pro-regenerative phenotype present in *dusp6* mutant hearts and supports an epistatic relationship where *foxm1* functions downstream of *dusp6* in heart regeneration. Next, we sought to determine whether inhibition of Erbb2, the Nrg1 receptor, would reduce the number of *foxm1*^+^ cardiomyocytes within the injury border zone. Previous studies have shown that the Erbb2 inhibitor AG1478 was sufficient to reduce cardiomyocyte proliferation by limiting downstream activity of the Nrg1/Erbb2/Ras/Mapk cascade (Gemberling et al., 2015; Missinato et al., 2018). Ventricular resections on WT and *erbb2st^61/+^* (referred to as *erbb2^m/+^*) adult zebrafish were performed and retro-orbital injections of DMSO or AG1478 were administered. At 7 dpa, fluorescence *in situ* hybridization revealed decreased *foxm1* staining in border zone cardiomyocytes from *erbb2^m/+^* treated with AG1478 compared to control WT hearts (Fig. 3I). Taken together, our data suggest that *foxm1* expression in cardiomyocytes is regulated by the Nrg1/Erbb2/Ras/Mapk pathway.

### Cell cycle progression gene transcripts are reduced in *foxm1^m/m^* hearts after injury

**Fig. 4.**
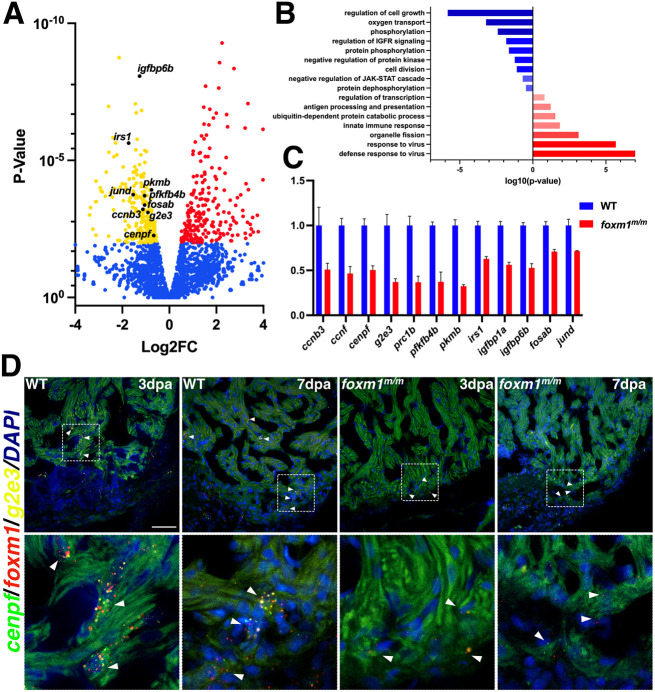
**Injured *foxm1^m/m^* hearts show reduced expression of G_2_ and M phase cell cycle genes.** (A) Representative volcano plot showing WT versus *foxm1^m/m^* 3 dpa gene expression from RNA-seq. Genes involved in cell cycle progression, insulin signaling, glycolysis and the AP-1 transcription factor family were significantly downregulated in the *foxm1* mutant hearts. Red and yellow points indicate increased and decreased expression, respectively. Blue represents genes with no significant change. (B) FAC (DAVID) confirmed decreased activity of cell division and insulin signaling, whereas innate immune response is increased. (C) qPCR validation on a subset of the genes with data represented as fold change normalized to WT 3 dpa expression+s.d. (D) Fluorescence *in situ* hybridization of *foxm1* (red) and the cell cycle genes *g2e3* (yellow) and *cenpf* (green) show co-expression in a subset of border zone cardiomyocytes in WT 3 dpa and 7 dpa hearts, whereas *foxm1*^+^/*g2e3*^+^/*cenpf*^+^ cardiomyocytes were rarely detected in the *foxm1* mutants. White arrowheads indicate representative *foxm1*^+^/*g2e3*^+^/*cenpf*^+^ cardiomyocytes within the injury border zone. Scale bar: 50 μm.

FOXM1 is known to activate transcription of G_2_/M phase proliferation genes crucial for the completion of mitosis (Lam et al., 2013; Laoukili et al., 2005). Therefore, transcriptome profiling of *foxm1* mutants during heart regeneration was performed. WT and *foxm1^m/m^* ventricles were collected after injury for RNA-seq, and 246 genes were significantly increased, whereas 159 genes were decreased, in *foxm1^m/m^* 3 dpa hearts (Fig. 4A; Table S3). We observed decreased expression of genes involved in cell cycle progression, insulin signaling, glycolysis and AP-1 transcription factors (Fig. 4A; Table S3). FAC revealed that cell division and insulin signaling were decreased, whereas immune responses were increased, in *foxm1^m/m^* 3 dpa ventricles (Fig. 4B; Table S4). qPCR validation confirmed that G_2_-phase genes (*ccnf* and *g2e3*) and M-phase genes (*ccnb3*, *cenpf* and *prc1b*) were decreased in *foxm1^m/m^* ventricles (Fig. 4C). In addition, genes involved in insulin signaling (*irs1*, *igfbp1a* and *igfbp6b*), glycolytic genes (*pfkfb4b* and *pkmb*) and AP-1 transcription factors (*fosab*, *fosb* and *jund*) were also decreased (Fig. 4C). These genes function in pathways known to be crucial for zebrafish heart regeneration, suggesting that Foxm1 may regulate their expression after injury (Beisaw et al., 2020; Fukuda et al., 2020; Honkoop et al., 2019).

To determine whether these genes were expressed in cardiomyocytes after injury, *in situ* hybridization on WT and *foxm1^m/m^* injured hearts was performed. *irs1* was highly expressed in WT myocardium but was decreased in *foxm1^m/m^* hearts at 3 dpa (Fig. S4A,B,E). Also, protein regulator of cytokinesis 1b (*prc1b*) was expressed in border zone cardiomyocytes at 3dpa but its expression was decreased in *foxm1^m/m^* hearts (Fig. S4C,D,F). Further, triple fluorescence *in situ* hybridization using *foxm1*, *g2e3* (G_2_ phase) and *cenpf* (M phase) revealed co-expression in cardiomyocytes near the injury border zone at 3 dpa and 7 dpa (Fig. 4D; Fig. S5A-C). These *foxm1*, *cenpf* and *g2e3* triple-positive cardiomyocytes were not detected in *foxm1^m/m^* hearts, suggesting that mutant cardiomyocytes were not progressing into the latter phases of the cell cycle (Fig. 4D). Moreover, the triple-positive cardiomyocytes were present within the injury border (Fig. S5D), suggesting that *foxm1* is required for border zone cells to cycle through G_2_ and M phases and identifying a proliferative subpopulation that activates post-injury. Taken together, these data suggest that *foxm1* regulates multiple pathways required for cardiomyocyte proliferation and that the loss of this transcription factor prevents the proliferative switch, thereby impairing cardiac regeneration.

### *cenpf* mutants exhibit increased cardiomyocyte binucleation

**Fig. 5.**
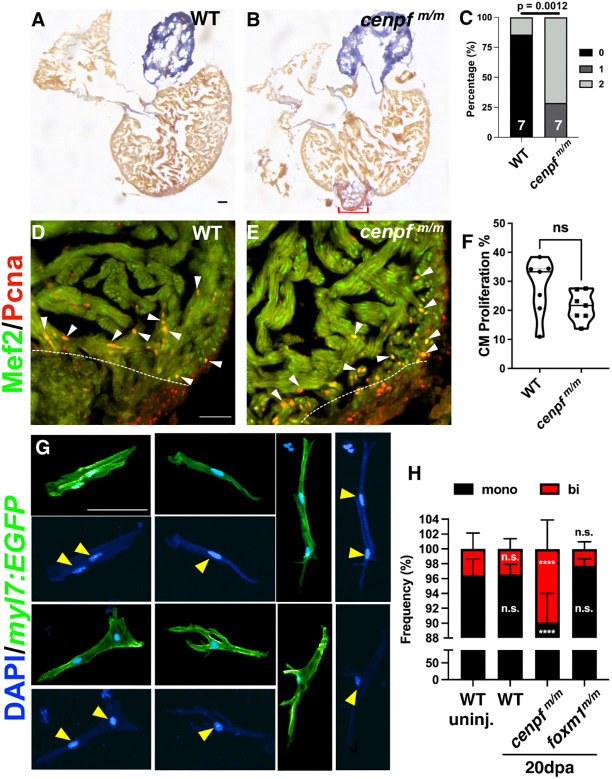
**Proportion of binucleated cardiomyocytes were increased in *cenpf^m/m^* hearts.** (A,B) WT (A; *n*=8) and *cenpf^m/m^* (B; *n*=8) 30 dpa hearts stained with AFOG to label fibrotic tissue. AFOG staining was used to label fibrin (red), collagen (blue) and muscle (orange-brown). Red bracket indicates fibrotic tissue. (C) Scar area was qualitatively scored: 0, no visible scar; 1, small amount of fibrosis with some collagen and fibrin stain; 2, medium to large amount of fibrosis with collagen and fibrin stain. (D-F) WT (D; *n*=7) and *cenpf^m/m^* (E; *n*=7) 7 dpa hearts showed no significant difference in cardiomyocyte proliferation index (F). White dotted lines represent the injury border and white arrowheads indicate Mef2^+^/Pcna^+^ cardiomyocytes. Truncated violin plot with each point representing an individual heart and these data represent three biological replicates. The middle line represents the median. (G,H) Individual cardiomyocytes were isolated from uninjured and 20 dpa hearts extracted from *Tg(myl7:EGFP)*, *Tg(myl7:EGFP)*; *cenpf^m/m^* and *foxm1^m/m^* zebrafish to count mononucleated versus binucleated cardiomyocytes. Representative images of mononucleated and binucleated cardiomyocytes are shown (G). Yellow arrowheads indicated nuclei from representative mononucleated and binucleated cardiomyocytes. The frequency (%) of WT uninjured (*n*=11 hearts; 1945 CMs; binucleation=3.52%), *cenpf^m/m^* uninjured (*n*=11; 3204 CMs; binucleation=7.24%), WT 20 dpa (*n*=11; 1858 CMs; binucleation=3.45%), *cenpf^m/m^* 20 dpa (*n*=12; 3072 CMs; binucleation=9.90%) and *foxm1^m/m^* 20 dpa (*n*=9; 2827 CMs; binucleation=2.28%) mononucleated (black) versus binucleated (red) cardiomyocytes was determined (H). Statistical analysis for AFOG was performed using the Fisher's exact test (C). CM proliferation index was calculated using the two-tailed, unpaired Student's *t*-test (F). Mononucleated versus binucleated counts used two-way ANOVA with Dunnett's multiple comparisons test (H). ****P<0.0001. n.s., not significant. Scale bars: 50 μm.

We next investigated the importance of *cenpf*, a known FOXM1 target gene. *Cenpf* plays a role in sister chromatid separation during mitosis (Liao et al., 1995) and is required for normal heart development (Dees et al., 2012, 2005). In mice, *Cenpf* mutant hearts possess thinner ventricular walls, reduced heart size (Dees et al., 2012) and decreased cardiomyocyte proliferation between P2 and P5 (Dees et al., 2005); however, its role in cardiac regeneration has not been characterized. We hypothesized that *cenpf* mutant hearts would display a similar phenotype to *foxm1* mutants after ventricular resection. The *cenpf^sa12296/sa12296^* line (referred to as *cenpf^m/m^*) contains a premature stop codon within Exon 6. This mutation is predicted to cause the loss of the entire C-terminal region and prevent Cenpf from interacting with spindle fibers during mitosis. *cenpf^m/m^* fish survived to adulthood with no cardiac abnormalities. After ventricular resection, *cenpf^m/m^* hearts retained large collagen/fibrin scars compared with WT controls at 30 dpa (Fig. 5A-C). Furthermore, fibrotic scars were still evident in *cenpf^m/m^* hearts as late as 60 dpa compared with controls (Fig. S6A-D). We next determined whether cardiomyocyte proliferation was affected by the loss of *cenpf*. In contrast to *foxm1^m/m^* hearts, no significant difference in the number of Pcna^+^ cardiomyocytes was detected in *cenpf^m/m^* hearts after injury (Fig. 5D-F). Fluorescence *in situ* hybridization revealed that expression of *foxm1*, *g2e3* and *prc1b* were unchanged in *myl7:EGFP*^+^ cardiomyocytes of *cenpf^m/m^* hearts when compared with controls (Fig. S6E,F). To further explore cardiomyocyte proliferation in *cenpf^m/m^* hearts post-injury, EdU incorporation assay was performed in injured WT and *cenpf^m/m^* zebrafish. At 7, 10 and 14 dpa, cardiomyocyte proliferation index in WT and *cenpf* mutant hearts showed no significant differences with staged matched hearts (Fig. S7). With prolonged fibrosis, unaltered expression of cell cycle genes and no changes in cardiomyocyte proliferation across multiple stages in *cenpf* mutant hearts post-injury, we postulated that a potential reason for the lack of new myocardium may be due to cytokinesis dysfunction in the regenerating cardiomyocytes.

During mammalian development, the heart primarily contains mononucleated diploid cardiomyocytes, but after P7 they either become mononucleated polyploid or binucleated (Bergmann et al., 2015; Mollova et al., 2013; Soonpaa et al., 1996). Unlike mammals, the majority of adult zebrafish cardiomyocytes remain mononucleated, with less than 5% of cardiomyocytes being binucleated (Gonzalez-Rosa et al., 2018; Patterson et al., 2017; Wills et al., 2008). Cytokinesis occurs after nuclei division and is characterized by cleavage furrow constriction and abscission. Binucleation is caused by cytokinetic failure, and it is theorized that these binucleated cardiomyocytes fail to re-enter the cell cycle following cardiac injury (Hesse et al., 2018; Leone et al., 2018; Liu et al., 2019). As Cenpf is known to associate to the kinetochore and spindle fibers before cytokinesis, we investigated whether the loss of *cenpf* increased cardiomyocyte binucleation after cardiac injury. We generated a *Tg(myl7*:*EGFP)*; *cenpf^m/m^* zebrafish to count nuclei within individual GFP^+^ cardiomyocytes. Mononucleated and binucleated cardiomyocytes were detected in these isolated cells (Fig. 5G). Binucleated cells made up 3.52% of the cardiomyocytes isolated from uninjured hearts and remained constant in WT 20 dpa hearts at 3.45%, indicating that no significant increase occurred post-injury (Fig. 5H). However, the percentage of binucleated cardiomyocytes increased by 3.68% in uninjured *cenpf^m/m^* hearts, which was double the amount detected in WT controls (Fig. 5H). After ventricular resection, the percentage of binucleated cardiomyocytes increased by 6.38% at 20 dpa in *cenpf* mutant hearts compared with controls (Fig. 5H). We also explored whether *foxm1^m/m^* hearts exhibited an increase in binucleated cardiomyocytes, but no significant increase was detected at 20 dpa (Fig. 5H), which indicates that this binucleation phenotype is specific to *cenpf^m/m^* hearts post-injury. Therefore, the loss of *cenpf* resulted in cardiomyocyte mitotic dysregulation during heart regeneration.

## DISCUSSION

**Fig. 6.**
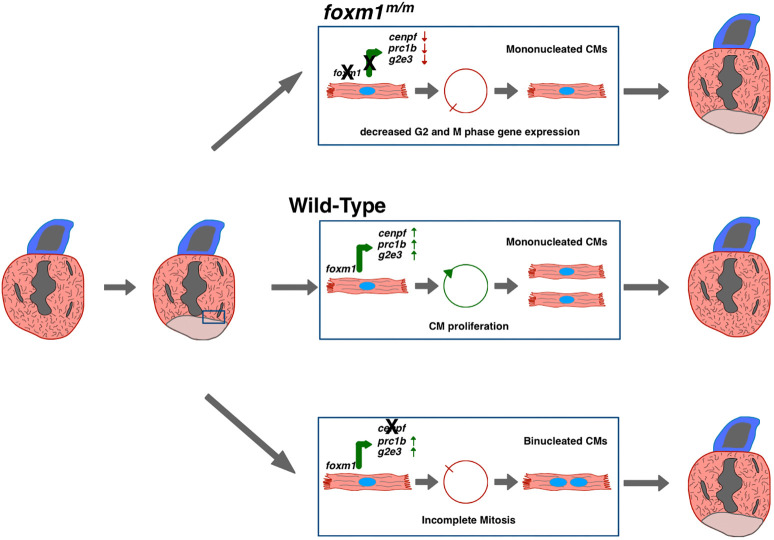
**A preliminary model indicating requirement of *foxm1* and *cenpf* expression in border zone cardiomyocyte cell cycle progression and mitosis.** In post-injury hearts, a subpopulation of border zone cardiomyocytes express *foxm1*. Foxm1 transcriptionally activates expression of cell cycle genes such as *cenpf*, *g2e3* and *prc1b* to promote regeneration of the lost myocardium (middle). In the absence of *foxm1* (top), induction of these G_2_ and M phase genes is decreased and border zone cardiomyocytes do not proliferate. In the absence of *cenpf* (bottom), cardiomyocytes may progress through the cell cycle until M phase, when Cenpf protein is required for chromatid separation. These dividing cells likely undergo cytokinetic dysregulation, which results in failed mitosis, generating binucleated cardiomyocytes.

In this study, we demonstrate that transcriptional regulation of late-stage cell cycle progression genes is essential for post-injury cardiomyocyte proliferation during heart regeneration. Foxm1 is necessary for cardiomyocyte proliferation through the induction of G_2_- and M-phase genes after injury (Fig. 6). Moreover, cell cycle progression genes, such as *cenpf*, are required for successful cardiomyocyte division after cardiac injury (Fig. 6).

Multiple factors can stimulate cardiomyocyte proliferation in adult zebrafish and neonatal mouse hearts after cardiac injury. Secreted ligands, such as Nrg1, Bmp2b, Tgf-β1, Pdgfbb, Igf2b and Fgf17b, produced by non-myocytes during cardiac injury contribute to this phenomenon (Bersell et al., 2009; Chablais and Jazwinska, 2012; Choi et al., 2013; Gemberling et al., 2015; Huang et al., 2013; Lepilina et al., 2006; Lien et al., 2006; Parodi and Kuhn, 2014; Wu et al., 2016; Zhao et al., 1998). Recent studies have highlighted the importance of transcription factors that regulate the expression of cell cycle genes in proliferating cardiomyocytes post-injury (Beauchemin et al., 2015; Beisaw et al., 2020; Sánchez-Iranzo et al., 2018; Sande-Melón et al., 2019; van Duijvenboden et al., 2019), and the loss of these factors prevents regeneration of the myocardium. Foxm1, a transcription factor required for proliferation in many different cell types and organs, is necessary for adult zebrafish cardiac regeneration as its loss prolonged fibrotic resolution. Beyond Foxm1 function in cardiomyocytes, other *foxm1*-expressing cells could contribute to the fibrosis phenotype we observed. A recent study by Goda et al. demonstrated that macrophage-specific deletion of *Foxm1* increased pro-inflammatory cytokine expression and promoted pulmonary fibrosis (Goda et al., 2020). This suggests that the macrophage profile in *foxm1^m/m^* hearts may have shifted from a pro-regenerative to pro-inflammatory phenotype, which would impede the removal of fibrotic tissue.

*Foxm1* is expressed in other cardiac cell types during mammalian development (Bolte et al., 2011; Ramakrishna et al., 2007). In the adult zebrafish heart, *foxm1* is not expressed, but is only induced in cardiomyocytes post-injury, suggesting a role in cardiac regeneration. FOXM1 regulates the transcription of G_2_/M-phase cell cycle genes through direct DNA binding at forkhead cis-regulatory element-containing promoters (Chen et al., 2013; Korver et al., 1998; Lam et al., 2013; Laoukili et al., 2005; Wang et al., 2002). This study revealed that the loss of *foxm1* resulted in decreased cardiomyocyte proliferation following injury. In addition to decreased expression of G_2_/M-phase cell cycle genes, expression of AP-1 transcription factors and glycolysis genes were also noted to be decreased in *foxm1* mutant hearts. The AP-1 transcription factor genes *junbb*, *fosl1a* and *fosab* are expressed in border zone cardiomyocytes and required for dedifferentiation and proliferation in regenerative cardiac models (Beauchemin et al., 2015; Beisaw et al., 2020; Wu et al., 2021). Glycolysis is the primary metabolic pathway used by border zone cardiomyocytes during proliferation in adult zebrafish and neonatal mice (Fukuda et al., 2020; Honkoop et al., 2019). In *foxm1^m/m^* resected hearts, we observed decreased expression of the rate-limiting glycolysis genes *pfkfb4b* and *pkmb*. Taken together, our study implies that Foxm1 could regulate cardiomyocyte dedifferentiation and the metabolic switch in proliferating cells, expanding on its known roles in cell cycle regulation.

Recent studies have shown that mutations within cell cycle-associated genes can increase the number of mononucleated polyploid and binucleated cardiomyocytes, which attenuate heart regeneration (Gonzalez-Rosa et al., 2018; Han et al., 2020; Hirose et al., 2019; Patterson et al., 2017). *ect2* encodes a protein required for cleavage furrow abscission during cytokinesis that was shown to reduce cardiomyocyte binucleation when overexpressed in neonatal rat cardiomyocytes (Liu et al., 2019). In contrast, inactivation of *etc2* via knockout or ectopic expression of a dominant-negative isoform caused both binucleation and polyploidization in neonatal rat and adult zebrafish cardiomyocytes (Gonzalez-Rosa et al., 2018; Liu et al., 2019; Windmueller et al., 2020). Cenpf functions in sister chromatid separation by associating to the kinetochores from prometaphase to early anaphase (Liao et al., 1995; Rattner et al., 1993). Silencing Cenpf disrupts chromosome alignment and can cause cytokinetic failure (Holt et al., 2005), which increases the incidence of binucleation during cell division. In this study, we observed prolonged retention of the fibrotic scar in *cenpf^m/m^* hearts. In addition to fibrotic resolution impairment, we observed an accumulation of binucleated cardiomyocytes after ventricular resection in *cenpf* mutants, indicating disrupted cytokinesis. In contrast, no increase of binucleated cardiomyocytes in *foxm1^m/m^* was detected, which we reason to be due to the lack of cell-cycle activity in the mutant hearts. This does not exclude the possibility of polyploidy as this phenomenon was reported in both cardiomyocytes and hepatocytes from *Foxm1* knockout mice (Korver et al., 1998; Krupczak-Hollis et al., 2004; Ramakrishna et al., 2007), and it may be possible that *foxm1^m/m^* cardiomyocytes fail to progress beyond G_2_ and develop a 4n ploidy. Alternatively, as *FoxM1* mRNA and protein levels typically increase at the entry of S phase (Laoukili et al., 2008), it is also possible that *foxm1^m/m^* cardiomyocytes fail to progress beyond the G_1_/S checkpoint and prematurely exit from the cell cycle without doubling their DNA content and remain as mononucleated diploid cells that exhibit a quiescent phenotype. Additional experiments will determine whether premature cell cycle exit at the G_1_/S checkpoint or accumulation of 4n cardiomyocytes are reasons for the lack of proliferation in *foxm1* mutant hearts after cardiac injury.

Overall, we demonstrate that *foxm1* and *cenpf* are expressed in a specific subpopulation of regenerating cardiomyocytes at the injury border. Disrupting the Foxm1-Cenpf axis reduced cardiomyocyte proliferation and mitosis via decreased G_2_/M cell cycle gene expression. These results illustrate the importance of a specific transcription factor, Foxm1, in cardiomyocyte proliferation, and these studies could assist in designing strategies to improve regeneration in the adult mammalian heart (Cheng et al., 2017).

## MATERIALS AND METHODS

Zebrafish lines, antibodies, chemicals and reagents from suppliers and catalogue numbers are listed in Table S5.

### Zebrafish maintenance, ventricular amputation, and retro-orbital injections

All zebrafish experiments and protocols were performed according to protocols approved by the Institutional Animal Care and Use Committee (IACUC) at the University of Pittsburgh in agreement with National Institutes of Health (NIH) guidelines. Adult (6- to 18-month-old) WT AB*, *Tg(myl7:EGFP)^twu34^* (Huang et al., 2003), *Tg(wt1b:EGFP)^li1^* (Perner et al., 2007), mutant *dusp6^pt30a^* (Missinato et al., 2018), *erbb2st^61/+^* (Lyons et al., 2005), and mutant *cenpf^sa12296^*, *foxm1^sa10708^*, *hmmr^sa12528^* and *cenpf^sa12296^* were acquired from the Zebrafish International Resource Center (ZIRC) (Kettleborough et al., 2013). DNA was isolated using Proteinase K (10 mg/ml) denaturation following adult tail fin clips for genotyping assays. PCR genotyping for *dusp6^pt30a pt30a^*, *foxm1^sa10708/sa10708^*, *cenpf^sa12296/sa12296^* and *hmmr^sa12528/12528^* (Table S6) was performed and products were digested with the restriction enzymes Cla1, Sal1, Spe1 or Age1, respectively. TaqMan SNP assays (Thermo Fisher Scientific) using custom probes for *cenpf*, *foxm1* and *erbb2* (Table S6) were also used to identify mutant single-nucleotide polymorphisms.

Ventricular amputation was performed as previously described (Poss et al., 2002), with ∼20% of the ventricle apex being resected. Zebrafish were returned to the aquarium for standard feeding and husbandry before hearts were extracted for DNA, RNA or histology at specific time points.

Retro-orbital injections were performed as previously described (Missinato et al., 2018). For 5-ethynyl-2′-deoxyuridine (EdU) labeling, EdU (Thermo Fisher Scientific) was dissolved in 100% DMSO (Sigma-Aldrich) and diluted to 3 mg/ml in 1× PBS. For EdU labeling, 3 μl of 50% DMSO in 1× PBS (vehicle) or 3 mg/ml EdU was injected into the left eye of the zebrafish 1 day before extraction to allow for 24 h of circulation. Hearts were extracted at the desired endpoints and were fixed in 4% paraformaldehyde (PFA) for immunofluorescent (IF) staining. To inhibit Erbb2, 25 μM AG1478 (Sigma-Aldrich) in 50% DMSO/PBS was prepared. Then 3 μl of control 50% DMSO/PBS (vehicle) or 25 μM AG1478 was injected into the eye of the adult zebrafish every 24 h until 3 dpa when the hearts were extracted.

### RNA-seq sample preparation and data analysis

Total RNA was extracted from uninjured and 3 dpa ventricles from WT, *dusp6^pt30a/pt30a^* and *foxm1^sa10708/sa10708^* adult zebrafish. A minimum of eight hearts were pooled together for each condition and RNA was isolated as previously described. For RNA-seq, 0.1-0.5 μg RNA for each condition was sent to the Genomics Research Core at the University of Pittsburgh and to Tufts University for library preparation and sequencing. The raw sequence reads were processed and mapped to the Zebrafish Reference Genome GRCz11 using CLC Genomics Workbench 20. Differentially expressed genes were calculated within the CLC software package and classified based on an FDR≤0.01 and Log2FC≥0.59 for uninjured versus 3 dpa experiment. For the *foxm1* mutant heart RNA-seq study, differential expression was based on FDR≤0.05 and Log2FC≥0.59. FAC was performed using The Database for Annotation, Visualization and Integrated Discovery (DAVID) (Huang da et al., 2009; Sherman et al., 2022).

### qPCR

For qPCR experiments, total RNA was extracted from uninjured and hearts at 3 dpa using Trizol (Invitrogen) and RNeasy micro kit (Qiagen). Eight hearts were pooled together for each condition. Between 0.5-1 μg total RNA was reversed transcribed with SuperScript Reverse Transcriptase (Thermo Fisher Scientific). qPCR was performed as previously described (Missinato et al., 2015). Primers for qPCR are listed in Table S6. The graphs (Figs 1C and 4C) show a representative example with the mean and standard deviation of the three technical replicates in one biological experiment. At least two independent biological replicates were performed.

### scRNA-seq data analysis

The mouse single cell data was generated previously (Li et al., 2019) and downloaded from GEO under the accession number GSE122403. Isolated cardiomyocytes from left ventricles were used for the analysis, including cell cycle phase annotation and gene expression analysis in Seurat V3 following a procedure as described previously (Li et al., 2019).

### AFOG fibrosis assay

For fibrosis assays, uninjured and injured hearts were collected in ice-cold 1× PBS and fixed in 4% PFA in PBS for either 2 h at room temperature (RT) or overnight at 4°C. Hearts were transferred into a sucrose gradient (10%, 20%, 30% sucrose in 1× PBS; ∼1 h for each solution) and stored in 30% sucrose in 1× PBS at 4°C overnight. Hearts were embedded in Surgipath Cryo-Gel (Leica Biosystems) the following day, and samples were sectioned on a Leica CM1850 cryostat at 14 µm and were dried overnight at RT. For fibrosis staining, we performed AFOG staining, which labels muscle as orange-brown, collagen as blue and fibrin as red, as previously described (Poss et al., 2002). Images were taken with a Leica MZ16 microscope and Q Imaging Retiga 1300 camera. Fibrotic tissue was defined as the combined area of blue (collagen) and/or red (fibrin) pixels within the ventricular apex following apical resection. Fibrotic area was semi-qualitatively scored as 0 (no fibrotic tissue detected at the ventricle apex), 1 (small amount of fibrosis with some collagen and fibrin stain) or 2 (medium to large amount of fibrosis with collagen and fibrin stain). This method is preferable to measuring the accumulation of collagen/fibrin in smaller areas due to the variability between individual sections. Multiple sections were analyzed per individual heart. A quantitative calculation was measured as follows: fibrotic area and ventricle area were represented as pixels and were converted into microns squared (μm^2^) by measuring the length (in pixels) of 1 mm using the 5× objective. Scar area was calculated by dividing the fibrotic area by the ventricle area. The average scar area (μm^2^) was determined using four sections per individual heart. All scoring and analysis were performed by an experimenter blinded to the individual animal's genotype.

### Immunofluorescent and EdU staining

For IF staining, slides were washed in PBS-T, followed by deionized water, and were permeabilized using 4% hydrogen peroxide in 100% methanol for 1 h. Slides were washed in deionized water, then PBS-T, and incubated in IF blocking buffer (200 μl sheep serum, 100 μl DMSO, 20 μl TritonX-100, and 9680 μl of PBS). Primary antibodies used for IF staining were rabbit polyclonal anti-Mef2 (Santa Cruz Biotechnology) and mouse monoclonal anti-Pcna (Sigma-Aldrich). Secondary antibodies were Alexa Fluor 488 goat anti-rabbit IgG peroxidase conjugate (Invitrogen) and Alexa Fluor 594 goat anti-mouse IgG (H+L) (Invitrogen). For full antibody details see Table S5. Slides were treated with DAPI (Thermo Fisher Scientific), washed in PBS and sealed using Aqua-PolyMount (Polysciences). Images were taken on a Zeiss 700 confocal microscope using a 20× objective. Image analysis was performed using ImageJ Fiji (NIH). For EdU Click-It chemistry, heart sections were permeabilized using 4% Triton X-100 in 1× TBS at room temperature followed by 1× TBS washes. For the Click-It Reaction solution, the following reagents were added to a 1.5 ml tube: 1× TBS (752 µl), 1 M Cu(II)SO_4_ (4 µl), Alexa Fluor 594 azide (144 µl; Thermo Fisher Scientific) and 1 M (+)-sodium-L-ascorbate (100 µl). Click-It Reaction solution was added to the heart sections and slides incubated in the dark for 30 min. Slides were washed in 1× TBS and incubated with blocking buffer (1% bovine serum albumin/5% sheep serum in 0.2% Triton X-100 in 1× TBS). For staining, primary antibody for rabbit anti-Mef2a/Mef2c (Abcam) was used with goat anti-rabbit Alexa Fluor 488 (Thermo Fisher Scientific) as secondary antibody. To label nuclei, DAPI was used, and slides were sealed using Aqua-PolyMount.

### Cardiomyocyte proliferation index

After immunofluorescent staining of heart sections with Mef2 and Pcna, the cardiomyocyte proliferation index (%) was calculated from the number of Mef2^+^/Pcna^+^ divided by the total number of Mef2+ cells. A total of four sections were counted per individual heart and a minimum of two biological replicates used in each experiment.

### RNAscope

RNAscope [Advanced Cell Diagnostics (ACD)] was performed on uninjured and injured (3, 7 and 14 dpa) hearts isolated from AB*, *Tg(myl7:EGFP)^twu34^*, *Tg(wt1b:EGFP)^li1^* and mutant *foxm1^sa10708^* adult zebrafish. Hearts were fixed in 4% PFA, transferred into a sucrose gradient (10%, 20%, 30%) the following day at 4°C before cryopreservation overnight. Tissue was embedded in Surgipath Cryo-Gel and sectioned at 14 µm. RNAscope probe hybridization, amplification and immunostaining were performed following the protocol provided by the manufacturer. ACD designed all the probes used in this study and they are listed in Table S5. For some sections, following the final wash step of the RNAscope probe hybridization protocol, immunofluorescent staining was performed to better visualize endogenous GFP from the transgenic lines using primary antibodies, chicken anti-GFP (Aves Labs). Secondary antibodies for immunostaining were fluorescein goat anti-chicken 488 (Aves Labs). For full antibody details see Table S5. Slides were treated with DAPI (1:500). Images were taken on a Zeiss 700 confocal microscope using a 40× water objective. Image analysis was performed using ImageJ Fiji (NIH).

### RNAscope quantifications

Using ImageJ software, individual channels were converted into 8-bit images and the threshold was set to minimize the background signal. A myocardium mask was generated from an 8-bit threshold image in the GFP channel using *Tg(myl7:EGFP)^twu34^* or the auto-fluorescent 488 channel from non-transgenic hearts. The myocardium mask was applied over the 8-bit image with an individual probe and particles were analyzed using the ‘Analyze Particles’ tool to count the number of individual probes within the myocardium mask. A total of four sections were imaged per heart. An alternative method used was to calculate the correlated total cell fluorescence (CTCF). In the channel of an individual probe, ten regions of interest (ROIs) of equal size were drawn around sections of myocardium that expressed probe within 50 μm from the injury border. The mean of five ROIs taken from areas lacking both myocardium and probe served as the background fluorescence. CTCF was calculated as ‘CTCF=Integrated Density – (area of selected cell×mean background fluorescence)’ to calculate probe fluorescence. For graphs, CTCF is referred to as ‘Fluorescence (arbitrary units; a.u.)’. A total of four sections were imaged per heart.

### Cardiomyocyte dissociation

We performed ventricular resections on *Tg(myl7:EGFP)^twu34^*, *Tg(myl7:EGFP)^twu34^*:*cenpf^m/m^* and *foxm1^m/m^* adult zebrafish and collected hearts at 20 dpa. For a crude dissociation, we used the assay previously described by Gonzalez-Rosa et al. (2018). Individual hearts were extracted and placed in ice-cold dissection buffer (0.3% bovine serum albumin and 20 mM glucose in 1× PBS) before removing the bulbous arteriosus and atrium from the ventricle. The ventricles were cut into smaller pieces, washed in fresh dissection buffer for 5 min and treated with digestion solution [20 mM glucose 10 mM BDM into 1× Trypsin-EDTA (Thermo Fisher Scientific)] for 15 min with gentle agitation. Digestion solution was removed, ventricles were washed with dissection buffer+BDM (0.3% bovine serum albumin, 20 mM glucose and 10 mM BDM in 1× PBS). Following these washes, ventricles were treated in a collagenase digestion solution [20 mM glucose and 10 mM BDM in 100% Accumax solution (EMD Millipore)] for 45 min with occasional agitation. Ventricles were pipetted in the collagenase digestion solution to release single cells and solutions were pelleted by centrifugation at 400 ***g*** for 5 min. Collagenase solution was removed without disturbing the pellet, and cells were fixed with 4% PFA. Cells were pelleted by centrifugation (400 ***g*** for 5 min), fixative was removed and cells were resuspended in 1× PBS. Single cell suspension was spread onto SuperFrost Plus microscope slides and dried overnight.

### Binucleation counts

For fixed single cell suspension, slides were permeabilized in 0.5% Triton X-100 in 1× PBS and incubated in blocking buffer (5% goat serum and 0.1% Tween 20 in 1× PBS) for 30 min. Chicken anti-GFP was used as primary antibody for transgenic hearts and rabbit anti-troponin I (Abcam) was used for non-transgenic *foxm1^m/m^* hearts. For full antibody details see Table S5. Slides with primary antibody were incubated at 4°C overnight. Slides were washed in 0.5% NP40 in 1× PBS. Fluorescein goat anti-chicken antibody or goat anti-rabbit (Thermo Fisher Scientific) were used as secondary antibody and slides were incubated for 2 h. Slides were washed in 0.5% NP40 in 1× PBS. All slides were treated with DAPI for 10 min and were immediately washed in 1× PBS before being sealed using Aqua-PolyMount. Images were taken on a Zeiss 700 confocal microscope using a 20× objective and ImageJ FIJI (NIH). GFP^+^ binucleated cardiomyocytes were manually counted along with all other GFP^+^ mononucleated cardiomyocytes and the mean percentage+s.d. of mononucleated and binucleated cardiomyocytes was represented in a stacked bar graph.

### Statistical analysis

Statistical analyses were determined using GraphPad Prism version 9.3. Statistical significance was analyzed by two-tailed, unpaired Student's *t*-test, one-way ANOVA, two-way ANOVA or Fisher's exact test. Data are shown as mean±s.d. or mean±s.e.m. *P*<0.05 was considered significant.

## Supplementary Material

10.1242/develop.201163_sup1 Supplementary information
