# Supplementary material for: Foxm1 regulates cardiomyocyte proliferation in adult zebrafish after cardiac injury
Source: Development. 2023 Mar 14;150(6):dev201163. doi: 10.1242/dev.201163 (PMC10108034; doi:10.1242/dev.201163)
Supplement: Supplementary information [file develop-150-201163-s1.pdf]

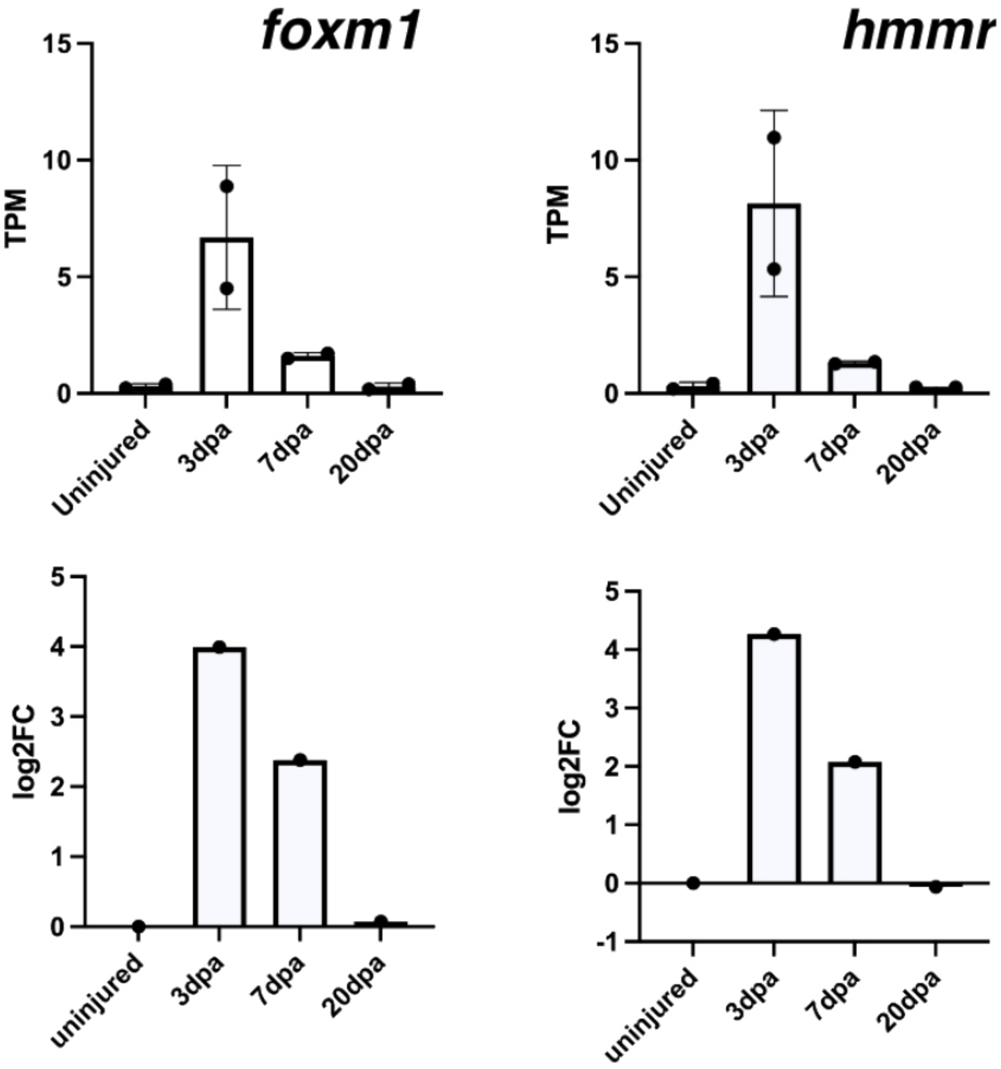

Fig. S1. RNA-Seq reveals expression of *foxm1* and *hmmr* increased after ventricular resection and decreases by 20dpa. Top panels show transcript per million (TPM) for *foxm1* and *hmmr* at the stages indicated. Bottom panels shop log2Fold Change (log2FC) *of foxm1* and *hmmr* compared to uninjured adult hearts.

Development • Supplementary information

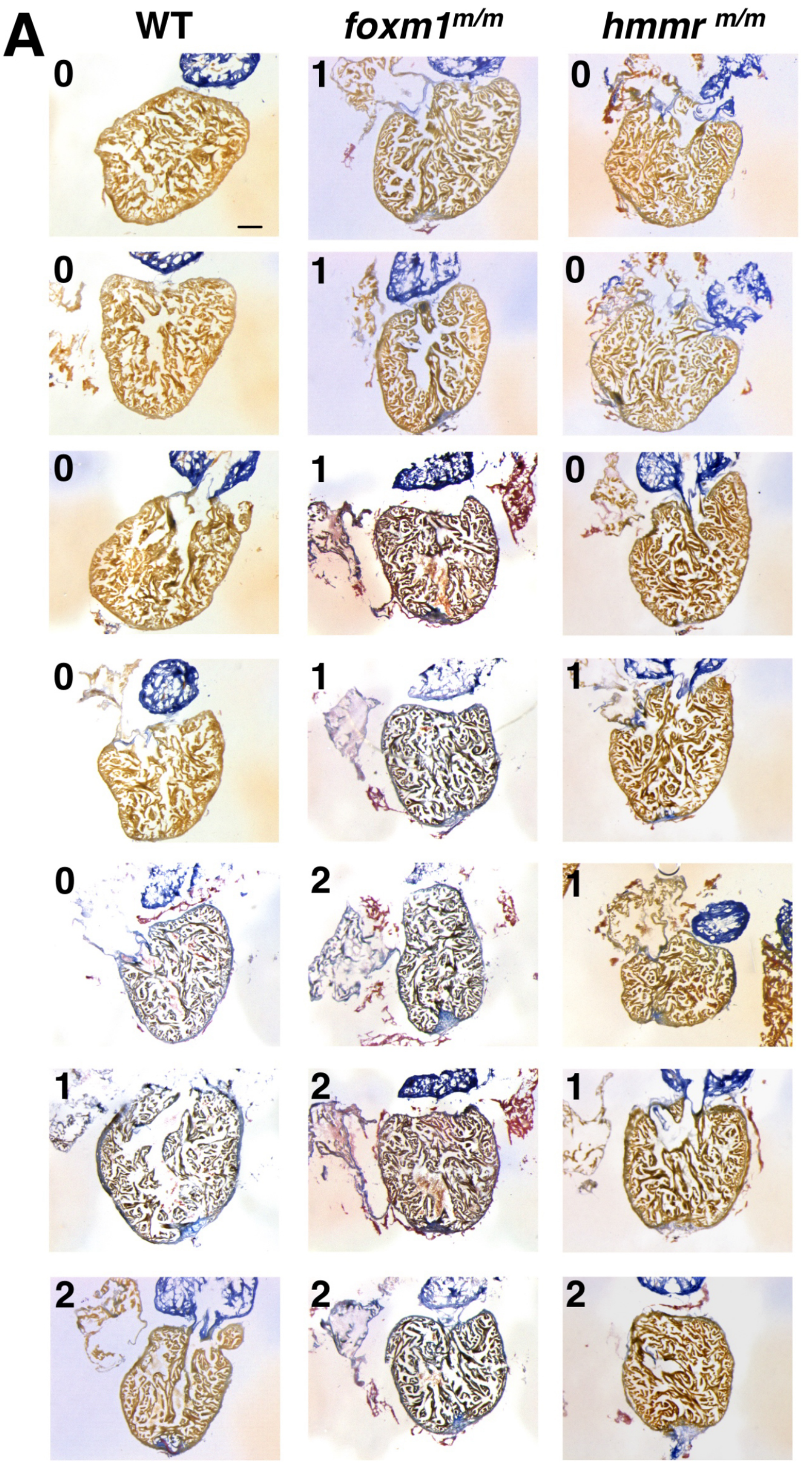
A
WT
foxm1 m/m
hmmr m/m
0
1
0
0
1
0
0
1
0
0
1
1
0
2
1
1
2
1
2
2
2

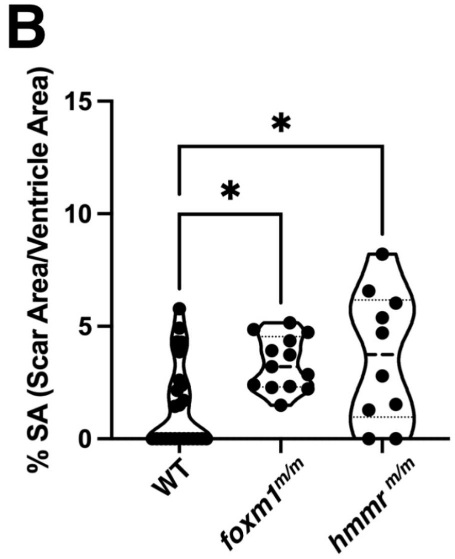
B
% SA (Scar Area/Ventricle Area)
15
10
5
0
*
*
WT
foxm1 m/m
hmmr m/m

Fig. S2. Representative AFOG images of WT 30dpa, *foxm1*$^{m/m}$ 30 dpa, and *hmmr*$^{m/m}$ 30 dpa hearts. Individual hearts from WT (n=22), *foxm1*$^{m/m}$ (n=13) and *hmmr*$^{m/m}$ (n=7) at 30dpa hearts were qualitatively scored as 0= no visible scar, 1= small amount of fibrosis with some collagen and fibrin stain, or 2= medium to large amount of fibrosis with collagen and fibrin stain. Representative images for individual hearts taken using a 5X objective are shown here (A). Scar area (SA, µm^2) was calculated as fibrotic area (µm^2) divided by ventricle area (µm^2). The average SA was determined using 4 sections per individual heart. Statistical analysis was calculated by one-way ANOVA with Dunnett's multiple comparison test as violin plots. P value: * = 0.0171 (WT vs *foxm1*$^{m/m}$) or 0.0111 (WT vs *hmm1*$^{m/m}$) **.** Scale bar = 50µm.

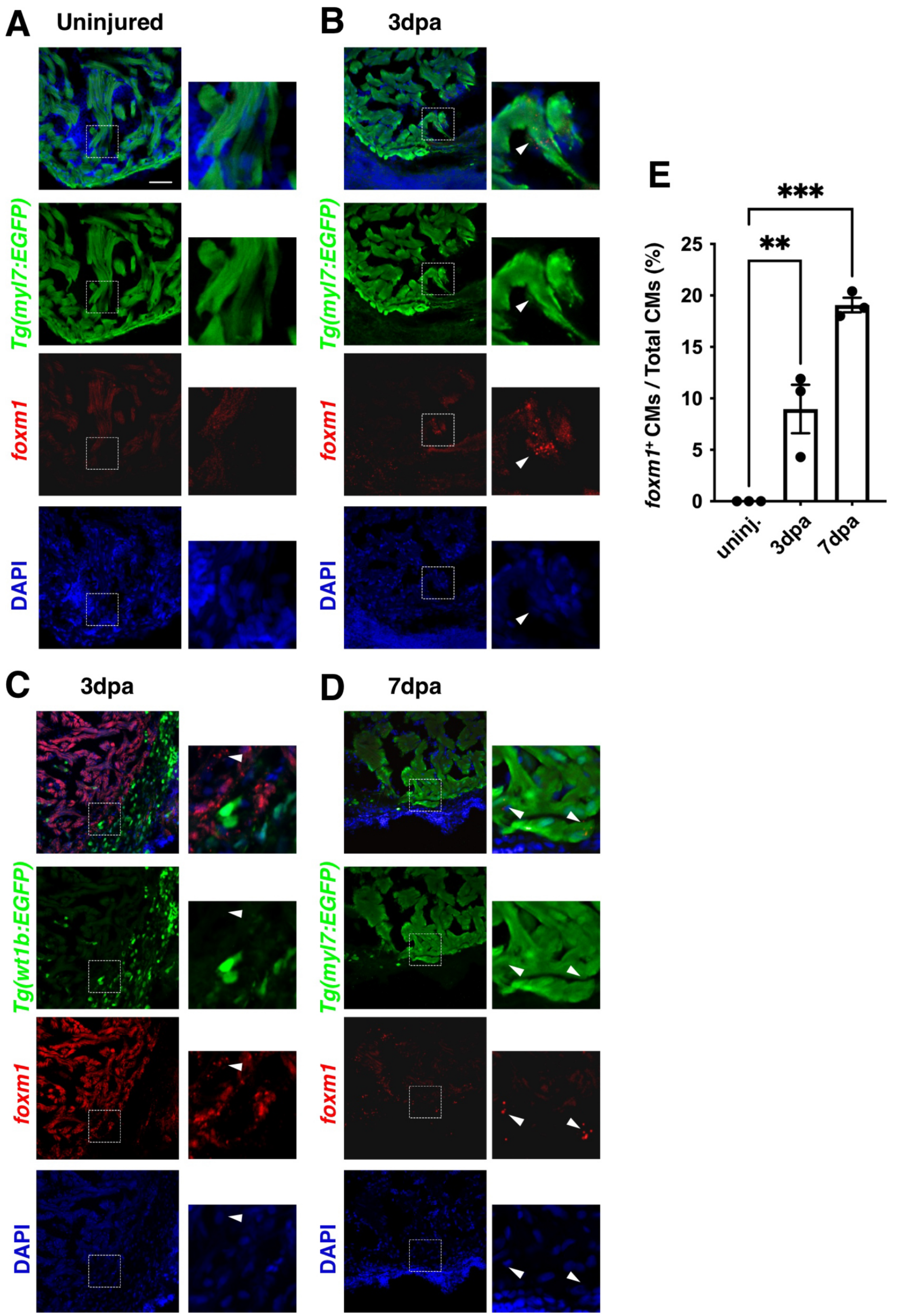

Development • Supplementary information

Fig. S3. *Foxm1* is predominantly expressed in the border zone myocardium after ventricular resection. Fluorescent *in situ* was used in *Tg(myl7:EGFP)* and *Tg(wtlb:EGFP)* hearts to determine the spatial expression of *foxm1* . *foxm1* is not expressed in the uninjured *Tg(myl7:EGFP)* heart (**A**; n = 3) but its expression is detected in GFP^+ cardiomyocytes at 3dpa (**B**; n = 3) and 7dpa (**D**; **n = 3**) in *Tg(myl7:EGFP).* In contrast, *foxm1* was not expressed in GFP^+ epicardial cells in *Tg(wtlb:GFP)* 3dpa hearts (**C**). White arrowheads indicate *foxml* mRNA within opp+ cells. The number of *foxm1*$^+$ GFP^+ cardiomyocytes increased after injury (**E**). Statistical significance was calculated by one-way ANOVA with Dunnett's multiple comparison test as either the mean + S.D as a bar graph. P value: ** = 0.0077, *** = 0.0002. Scale Bar = 50µm

Development • Supplementary information

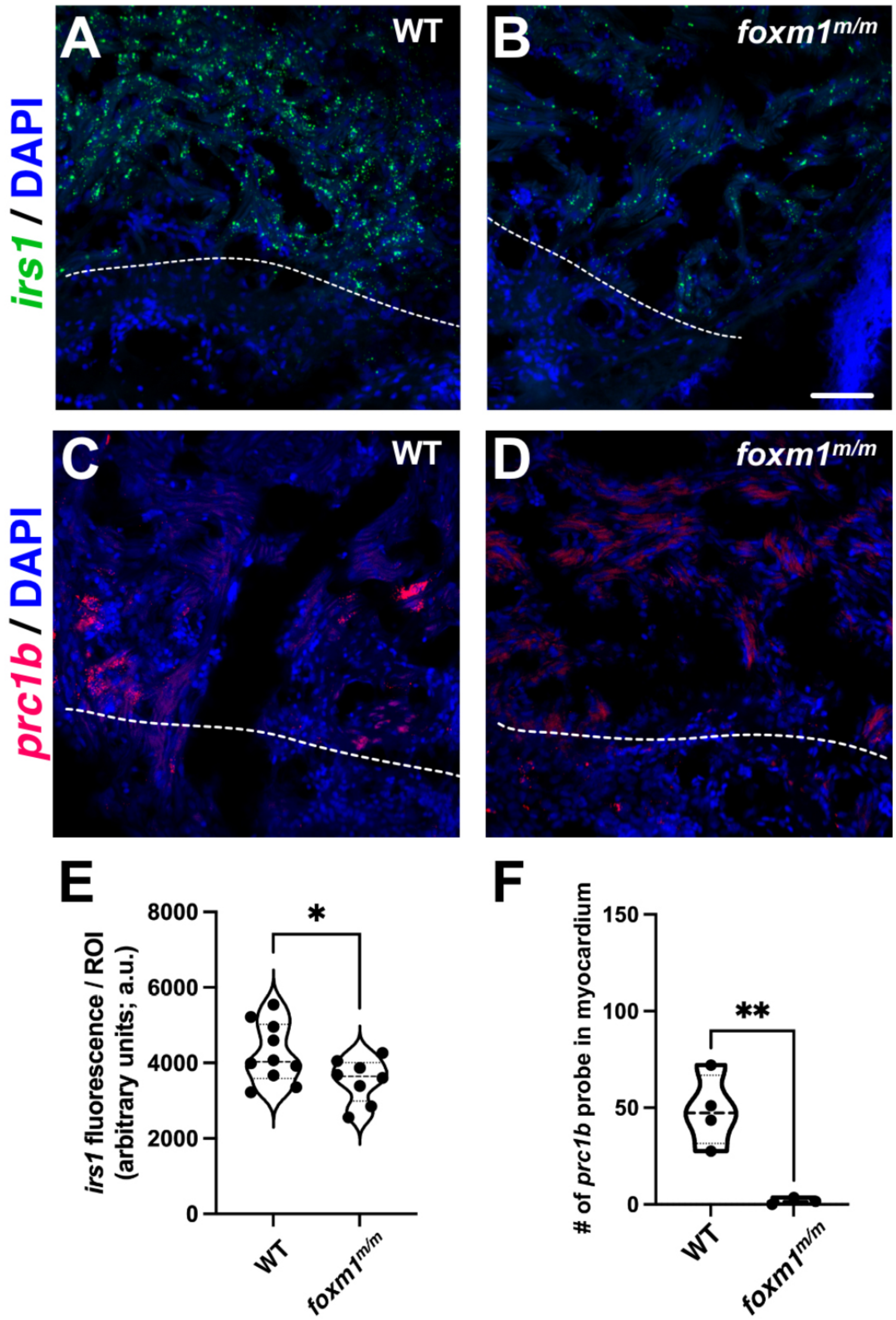

Development • Supplementary information

Fig. S4. *Irs1* and *prc1b* are decreased in *foxm1*$^{m/m}$ hearts. Fluorescent *in situ* was used to determine the spatial expression of *irs1* and *prclb. irs1* is highly expressed in WT at 3dpa in the myocardium **(A)**. In *foxm1*$^{m/m}$ hearts; *irs1* expression was decreased **(B)** in the myocardium. *Prec1b* expression is present in the border zone myocardium and clot in WT hearts **(C)**, but *foxm1*$^{m/m}$ hearts possessed reduced *prc1b* transcripts **(D)**. Probe fluorescence was calculated by generating ROIs near the injury border from individual sections **(E)** or by counting individual probes present within the myocardium of WT (n = 4) *and foxm1*$^{m/m}$ (n = 3) hearts **(F)**. Both *irs1* **(E)** and *prec1b* **(F)** expression was significantly decreased in *foxm1*$^{m/m}$ hearts. White dotted lines represent the injury border. Statistical significance was determined using the unpaired student's t-test. p values: * = 0.0477, ** = 0.0079. White dotted lines represent the injury border. Scale Bar = 50µm

Development • Supplementary information

Development: doi:10.1242/dev.201163: Supplementary information

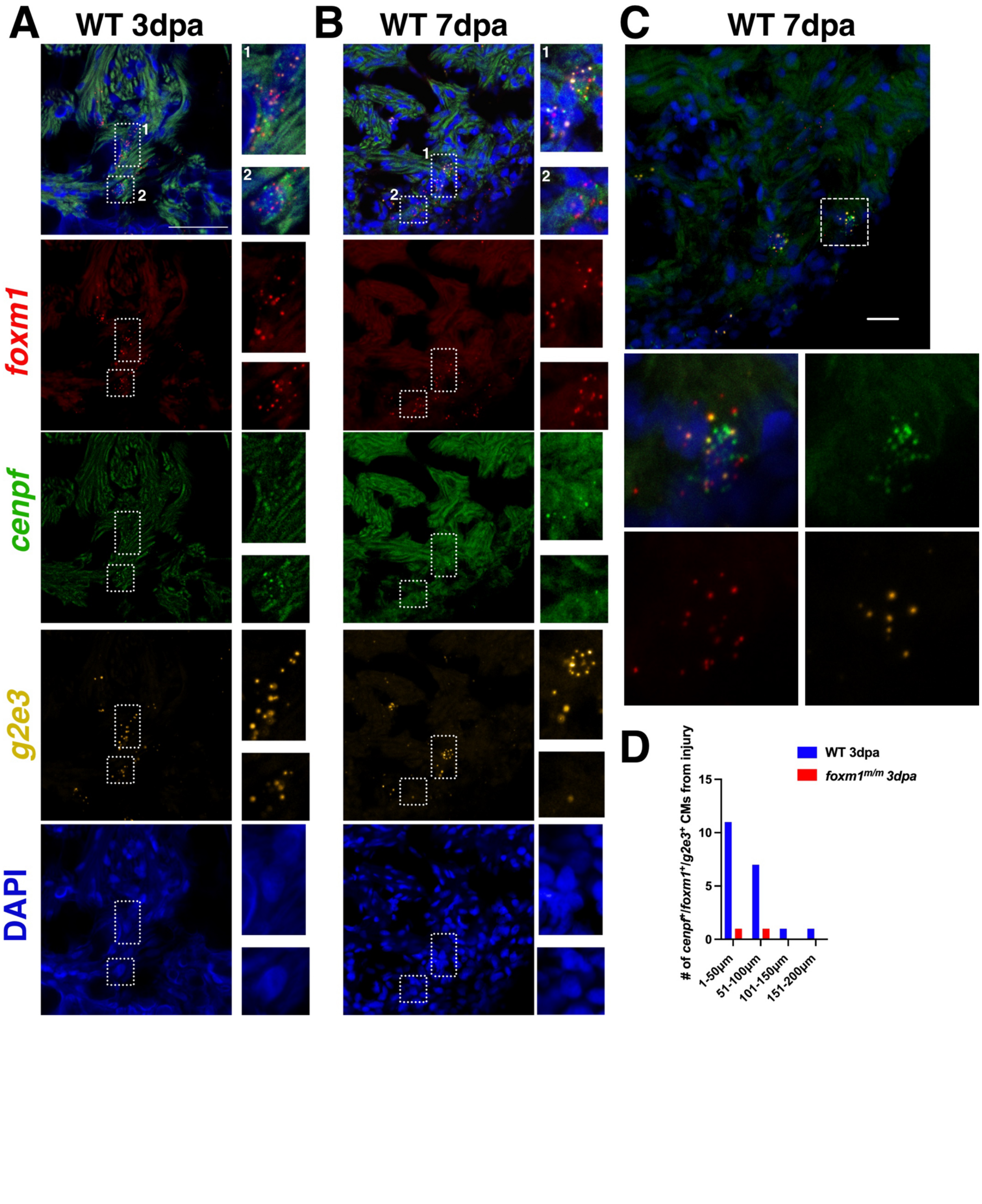

Development • Supplementary information

Fig. S5. *Foxm1⁺/g2e3⁺/cenpf⁺* cardiomyocytes are detected following ventricular resection. Fluorescence *in situ* hybridization for cell cycle genes was performed on WT 3dpa **(A)** and WT 7dpa **(B)** hearts. *Foxm1* (red; S- phase marker), *g2e3* (yellow; G2-phase marker), and *cenpf* (green; G2/M-phase marker) were used to identify cells progressing through specific stages of the cell cycle and are shown here in individual channels. Triple-positive cardiomyocytes required the expression of the probes near a DAPI$^+$ nuclei in the myocardium. Representative *foxm1$^+$ lg2e3$^+$/cenpf$^+$* cardiomyocytes are denoted in separate, smaller images. A 40X water objective image of WT 7dpa triple-positive cardiomyocytes **(C)** was split into individual channels to indicate *foxm1* (red), *g2e3* (yellow), and *cenpf* (green) expressing cardiomyocytes at a higher resolution. *Foxm1$^+$/ g2e3$^+$cenpf$^+$* cardiomyocytes were measured, and the majority of these cells were localized within 100mm from the injury and expression decreased distal to the wound **(D)**. Triple positive cardiomyocytes were counted in total from 5 hearts. Scale bar = 50µm.

Development • Supplementary information

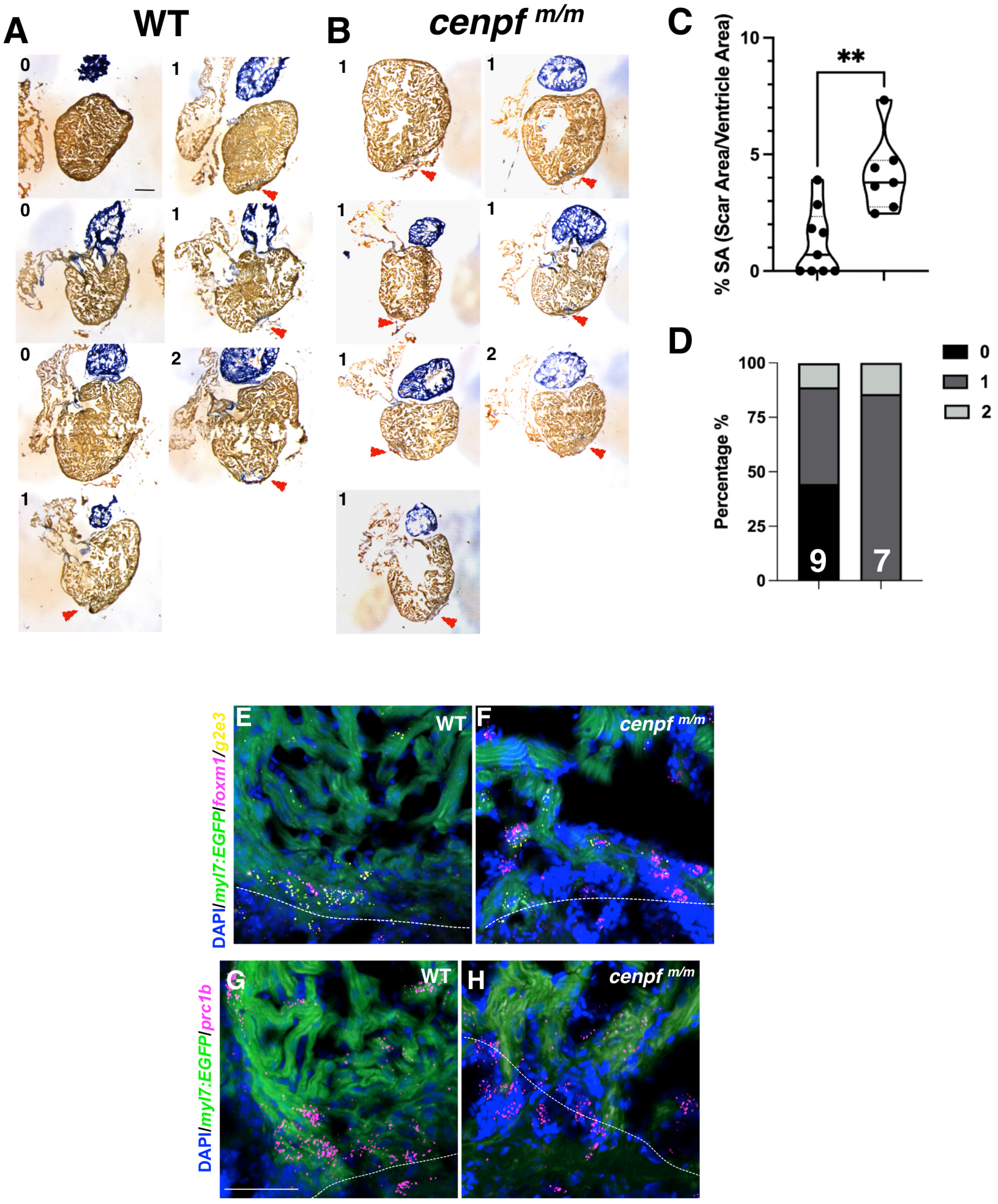

A
WT
B
cenpf m/m
C
% SA (Scar Area/Ventricle Area)
10
5
0
**
D
Percentage %
100
75
50
25
0
9
7
0
1
2
E
WT
F
cenpf m/m
DAPI/myl7:EGFP/foxm1/g2e3
G
WT
H
cenpf m/m
DAPI/myl7:EGFP/prc1b

Fig. S6. Characterization of *cenpf*$^{m/m}$ hearts. WT (**A;** n=9) and *cenpf*$^{m/m}$ **(B;** n=7) 60dpa hearts were stained with AFOG solution. Red arrowhead indicate scar tissue. Scar area (SA, µm^2) was calculated as fibrotic area (µm^2) divided by ventricle area (µm^2). The average SA was determined using 4 sections per individual heart. *Cenpf*$^{m/m}$ 60dpa hearts retained a collagen scar at the injury site that is significantly larger than WT controls **(C)**. Qualitative scoring of the fibrotic area was recorded as O= no visible scar, I = small amount of fibrosis with some collagen and fibrin stain, or 2= medium to large amount of fibrosis with collagen and fibrin stain **(D).** To determine if the prolonged fibrosis was the result of decreased cell cycle activity, fluorescent *in silu* probes were used on *Tg(myl7:EGFP)* and *Tg(myl7:EGFP); cenpf*$^{m/m}$ 7dpa hearts **(E-H).** *foxm1* (magenta) and *g2e3* (yellow) expression appeared unchanged between WT **(E)** and *cenpf*$^{m/m}$ **(F)** 7dpa hearts which indicates earlier cell cycle activity was unaltered. *Prc1b* levels were also unchanged at 7dpa in WT **(G)** and *cenpf*$^{m/m}$ **(H)** hearts. Dotted white lines were used to indicate the clot outline. All fluorescent *in situ* images were taken using a 40X water objective. Statistical significance was calculated using the unpaired student's t-test and represented using a violin plot. P value: ** = 0.0018. Scale bar = 50µm.

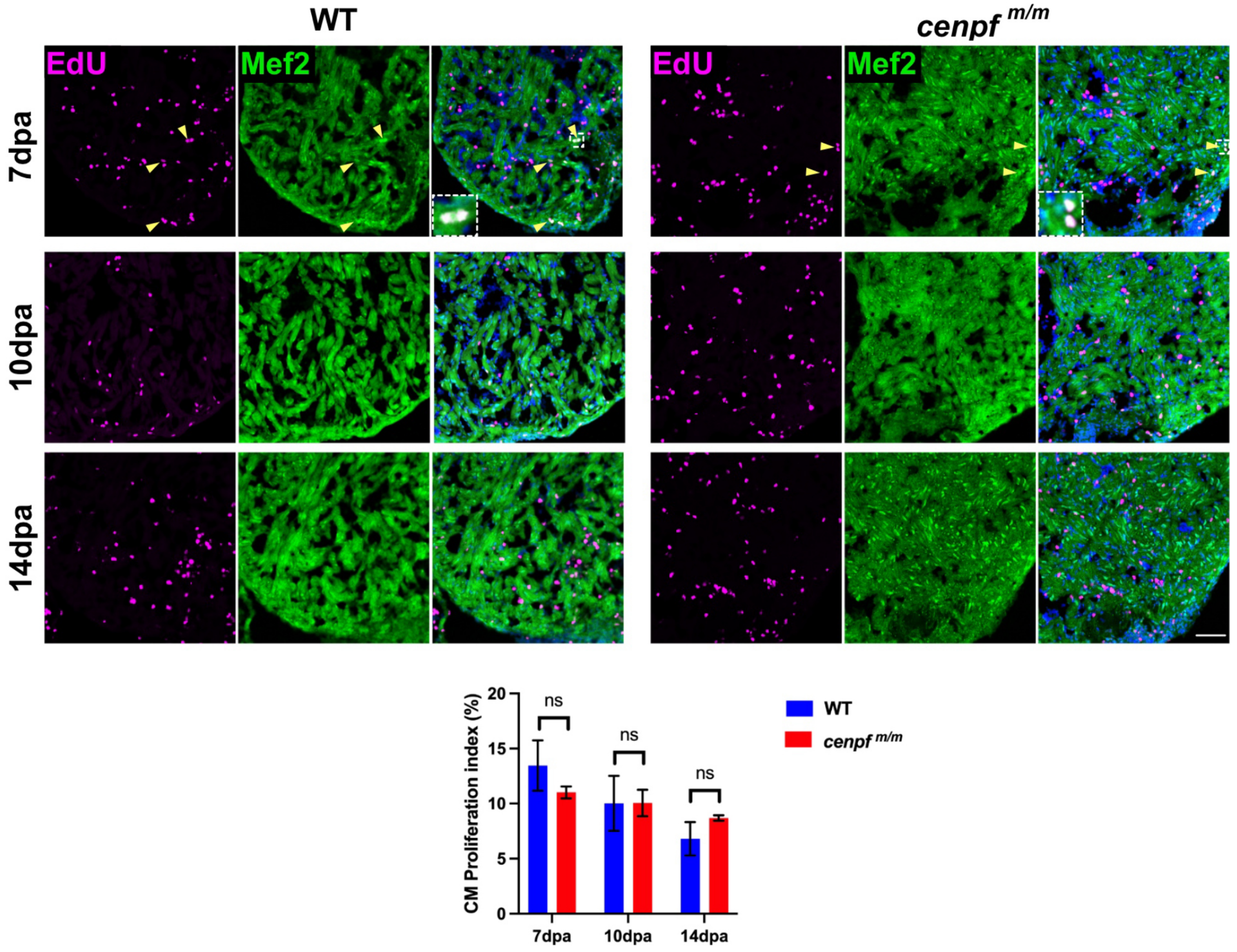

Fig. S7. Cardiomyocyte proliferation in *cenpf* mutants shows no significant decrease compared to WT hearts following EdU injection. Ventricular resected WT and *cenpf*$^{m/m}$ adult zebrafish at 5dpa, 8dpa, and 12dpa were injected with EdU over two days prior to heart extraction. Hearts were extracted at 7dpa, 10dpa, and 14dpa, and immunofluorescent staining for EdU and Mef2 was performed. The cardiomyocyte proliferation index was calculated as the number of EdU^{+}/Mef2^{+} cardiomyocytes divided by total CM number in the field of view. *cenpf*$^{m/m}$ 7dpa (n=3), 10dpa (n=4), and 14dpa (n=3) hearts showed no significant changes in cardiomyocyte proliferation compared to WT 7dpa (n=4), 10dpa (n=4), and 14dpa (n= 4) hearts. Yellow arrowheads indicate representative examples of EdU^{+}Mef2^{+} cardiomyocytes. Statistical analysis was performed by two-way ANOVA with Sidak's multiple comparisons test. All images were taken at 20X. Scale bar = 50µm.

Development • Supplementary information

Table S1. Excel document with listings of genes identified as increased or decreased at 3, 7 and 20dpa.

Click here to download Table S1

Table S2. Excel document showing DAVID analysis of GO terms and list of genes associated with increased expression at 3dpa compared to uninjured hearts.

Click here to download Table S2

Table S3. Excel document with listing of genes identified as decreased or increased in *foxm1* mutant hearts compared to WT at 3dpa.

Click here to download Table S3

Table S4. Excel document showing DAVID analysis of GO terms and list of genes associated with decreased expression in the foxm1 mutant heart at 3dpa compared to WT hearts at 3dpa.

Click here to download Table S4

Table S5. List of reagents used in this study with supplier information, catalogue number and dilutions used.

Click here to download Table S5

Table S6. List of primers used in the study for genotyping or for qPCR experiments.

Click here to download Table S6
