# Supplementary material for: Foxm1 regulates cardiomyocyte proliferation in adult zebrafish after cardiac injury
Source: Development. 2023 Mar 14;150(6):dev201163. doi: 10.1242/dev.201163 (PMC10108034; doi:10.1242/dev.201163)
Supplement: Supplementary file 1 [file develop-150-201163_review_history.pdf]

Foxm1 regulates cardiomyocyte proliferation in adult zebrafish after cardiac injury

Daniel A. Zuppo, Maria A. Missinato, Lucas Santana-Santos, Guang Li, Panayiotis V. Benos and Michael Tsang
DOI: 10.1242/dev.201163

Editor: Kenneth Poss

Review timeline

Original submission:	26 July 2022
Editorial decision:	31 August 2022
First revision received:	7 December 2022
Editorial decision:	9 January 2023
Second revision received:	4 February 2023
Accepted:	13 February 2023

Original submission

First decision letter

MS ID#: DEVELOP/2022/201163

MS TITLE: Foxm1 drives cardiomyocyte proliferation in adult zebrafish after cardiac injury

AUTHORS: Daniel A Zuppo, Maria Azzurra Missinato, Lucas Santana-Santos, Guang Li, Panagiotis Benos, and Michael Tsang

I have now received all the referees' reports on the above manuscript, and have reached a decision. The referees' comments are appended below, or you can access them online: please go to BenchPress and click on the 'Manuscripts with Decisions' queue in the Author Area.

As you will see, the referees express interest in your work but have some significant criticisms and recommend a substantial revision of your manuscript before we can consider publication. I agree that this work is interesting and has potential to implicate a new factor in zebrafish heart regeneration. If you are able to revise the manuscript along the lines suggested, which is likely to involve further experiments, I will be happy to receive a revised version of the manuscript. Your revised paper will be re-reviewed by one or more of the original referees, and acceptance of your manuscript will depend on your addressing satisfactorily the reviewers' major concerns. I will note that I agree generally with major comment number #2 by Reviewer #2 in feeling that the histological or quantified evidence of the foxm1 mutant defect as shown may not be convincing to readers. While you might try to measure scar percentage in AFOG-stained hearts of varying sizes and initial injuries, my recommendation is to display representative images of sections from each animal used for quantification in Fig. 1G-I in a supplemental figure, and to consider a semi-quantitative method that grades injuries like my group has used for several years.

Please also note that Development will normally permit only one round of major revision. If it would be helpful, you are welcome to contact us to discuss your revision in greater detail. Please send us a point-by-point response indicating your plans for addressing the referee’s comments, and we will look over this and provide further guidance.

© 2023. Published by The Company of Biologists under the terms of the Creative Commons Attribution License (https://creativecommons.org/licenses/by/4.0/).

Please attend to all of the reviewers' comments and ensure that you clearly highlight all changes made in the revised manuscript. Please avoid using 'Tracked changes' in Word files as these are lost in PDF conversion. I should be grateful if you would also provide a point-by-point response detailing how you have dealt with the points raised by the reviewers in the 'Response to Reviewers' box. If you do not agree with any of their criticisms or suggestions please explain clearly why this is so.

Reviewer 1

Advance summary and potential significance to field

In this study, Zuppo and colleagues identified the transcription factor foxm1 as a requirement for cardiomyocyte proliferation during adult zebrafish heart regeneration. By conducting RNA-seq in uninjured and 3 dpa WT and dusp6 mutant ventricles, they observed enhanced foxm1 expression after cardiac injury at 3 dpa. They also re-evaluated a published scRNA-seq dataset of embryonic mouse hearts, which demonstrated Foxm1 expression in cycling murine cardiomyocytes. foxm1m/m revealed declined cardiomyocyte proliferation at 7 dpa and defective myocardial recovery with enhanced fibrosis at 30 dpa in comparison to wild-type ventricles. Further, foxm1 was predominantly expressed in border zone cardiomyocytes and RNA-seq analysis of wild-type and foxm1m/m ventricles at 3 dpa uncovered decreased expression of cell cycle progression, insulin signaling, glycolysis, and AP-1 factors, implicating foxm1 as a regulatory factor in cardiomyocyte division. Moreover, this RNA-seq dataset identified diminished expression of cenpf in foxm1m/m ventricles in comparison to wild-type at 3 dpa. Examination of cenpf mutants demonstrated large fibrotic scars and increased cardiomyocyte binucleation at 30 dpa. Overall, the authors demonstrate the requirement of foxm1 and cenpf for effective cardiac regeneration; this will contribute to further findings and help isolate regulatory differences in zebrafish and mammalian injury responses in cardiomyocytes that could potentially be utilized to enhance regeneration in humans.

Comments for the author

Major Concerns/Questions:

1. The authors chose 3 dpa as the key time point for early zebrafish regeneration after ventricle resection to collect hearts for RNA-seq, but do not disclose their reasoning for this time point except the description “Cardiomyocytes respond to induced stimuli at 3 days post-amputation (dpa) and begin to dedifferentiate and divide”. It would be helpful to readers to include why this time point not peak time like 7 dpa was chosen.

2. The combination of the RNA-seq data from both WT and dusp6 mutant hearts is not explained. Was there no difference in DGE between WT and dusp6 mutant hearts at the time points observed (no injury and 3 dpa)?

3. In results subsection “Identification of mitotic genes involved in cardiac regeneration section” paragraph 1, the use of cluster in the phrase “Many genes identified in this cluster” is somewhat confusing. I infer that the authors are referring to the genes that were significantly enhanced after cardiac injury at 3 dpa, and suggest rephrasing to make the sentence clearer.

4. In qPCR to validate RNA-seq data in Results “Identification of mitotic genes involved in cardiac regeneration section”, are WT and dusp6 mutant hearts combined as with RNA-seq or are just WT hearts used to confirm RNA-seq results? Further, as hmmr is selected as an interesting candidate, hmmr gene expression should also be included in the representative qPCR in Fig. 1C.

5. In the supplementary Figure 1 A-D, the authors need to include the images of individual myl7:GFP or wt1b:GFP fluorescence and foxm1 fluorescence in addition to the image showing both signals. It is hard to see foxm1 red fluorescence at 3 and 7 dpa due to strong myl7+ green signals. Additionally, it appears as though 3 dpa foxm1 expression in the wt1b:GFP/foxm1 image is strongly expressed throughout the myocardium and not just in border zone cardiomyocytes, is this due to overexposure or high signal background for in situ foxm1 signals that are very different from myl7:EGFP/foxm1 images?

© 2023. Published by The Company of Biologists under the terms of the Creative Commons Attribution License (https://creativecommons.org/licenses/by/4.0/).

6. Does foxm1m/m influence myocardial regeneration at 30 dpa? Although the authors observed a decline during the peak time point of CM proliferation at 7 dpa, it would be necessary to compare myocardial recovery in wild-type and foxm1m/m at 30 dpa as the direct evidence to link the requirement of foxm1 for cardiomyocyte proliferation.

7. which cell types are these nrg1+ cells observed in Figure 3A and B, are from epicardial-derived cells? Other cell types, like Treg cells, also express nrg1 after cardiac injury in zebrafish (Hui et al., 2017 PMID: 29257949).

8. For supplementary Figure 2 A and B, there appears to be little to no difference in the representative figures shown between WT and foxm1m/m. The authors need to measure the signal intensity for irs1 signals at 3 dpa in the border region in WT and foxm1m/m. Also, it would be useful to include lower magnification images of the hearts, to better understand where foxm1 is expressed.

9. Can the authors include the data to show foxm1+ cardiomyocytes are proliferating cells by combine foxm1 in situ hybridization and MEF2/PCNA staining or EdU incorporation?

10. Figure 4D: it is very hard to see the triple positive signals for in situ foxm1, cenpf, and g2e3. There appears to be high green background from cenpf fluorescence signals, and the yellow color for g2e3 is difficult to observe. A different color will be used to represent g2e3 in Figure 4D. Also, it would be beneficial to include here or in the supplement, images of each in situ signal and then the image of all 3 signals together with DAPI. Additionally, the placement of the white arrowheads for the "triple positive" cells is somewhat ambiguous, they need to be placed closer to cells. It looks like most of the white arrowheads for foxm1m/m image at 3 dpa and 7 dpa are not labelling a "triple positive" cell, I do not see yellow signals for g2e3 in the bottom left cell labelled by arrowhead at 3 dpa or in any of the cells labelled by arrowhead at 7 dpa. It would be better to use a different color to label example cells that are not "triple positive" in foxm1m/m and use white coloring for "triple positive" cells and explain what arrowheads/colors are labeling in the figure legend.

11.The authors proposed that foxm1 drives the expression of cell cycle genes and promote cardiomyocyte proliferation. They showed g2-phase gene ccng2 is decreased in foxm1 mutant in Fig.4C. However, ccng2 was reported to negatively regulate cell proliferation (Zimmermann et al., Cell Cycle 2016). The authors need to explain or discuss.

12. The authors showed loss of foxm1 decreases cardiomyocyte proliferation and enlarged scar deposition. As foxm1 is proposed to regulate cardiomyocyte cycling, the authors need to explain or discuss why loss of foxm1 impairs fibrotic resolution?

13. Figure 5G,H. Quantification of the myocardium prc1b in situ signals in WT and cenpfm/m hearts would be beneficial, and lower magnification images of the injured hearts will be included. The representative image of prc1b signals in cenpfm/m ventricles should show less of the injury site and more of the border zone myocardium like in the WT figure in panel G for a better comparison between the wild-type and mutant ventricles.

Minor:
1. In the introduction, pg. 1 paragraph 2: insert "for" between required and ATP to update sentence as follows: "Mature cardiomyocytes use fatty acid oxidation to generate substrates required for ATP generation via oxidative phosphorylation"

2. In the introduction, pg. 2 paragraph 3: provide full name of AP-1 (activator protein-1) before using the abbreviated name

3. Figure 2: Please provide name of the myocardium reporter used to the left of Fig.2 A and A', is it myl7:GFP? Please add for clarity in figure 2 and add in the figure 2 legend.

4. Figure 2 Legend does not match with the Figure names in Figure 2 and the text of the manuscript: WT is labeled as D-E but should be D-D' and foxm1m/m is labelled as F-G but should be

© 2023. Published by The Company of Biologists under the terms of the Creative Commons Attribution License (https://creativecommons.org/licenses/by/4.0/).

E-E', label H should be changed to F. There is also an empty box symbol before 0.0001 p-value in figure legend, this should be removed or the appropriate symbol inserted/fixed.

5. Supplementary Figure S1 Legend: What do the white and yellow arrowheads label?

6. Figure 3A: Add a box to indicate the region of the ventricle highlighted in Figure 3B

7. Figure 5 Legend: There is a box symbol before 0.0001 in description of p-values, should be removed/fixed.

8. Figure 5I: May be helpful for readers if panels are split into 2 sections with example images of mononucleated and binucleated cardiomyocytes separated and labeled.

9. Figure 6 legend: There is no Figure 6, this legend/info should be removed.

10. Supplementary Figure S2 legend: The labeling throughout the legend is off and does not match the manuscript text and Supp. Fig. S2. There are no uninjured hearts shown in the figure so mention of these in the legend should be removed. Needs to be changed so that (A) is 3 dpa WT hearts with irs1, (B) is 3 dpa foxm1m/m with irs1, (C) is WT 3 dpa hearts with prc1b and (D) is 3 dpa foxm1m/m with prc1b.

Reviewer 2

Advance summary and potential significance to field

This manuscript from Zuppo and colleagues describes the role of the forkhead transcription factor foxm1 during zebrafish heart regeneration. In response to ventricular resection, foxm1 levels increase 10-fold, and foxm1 mutant animals retain some scar tissue at 30 days post-injury. This regenerative defect is due at least in part, to a reduction in cardiomyocyte proliferation at 7 days post-injury. Transcriptional profiling of foxm1 mutant hearts revealed a reduction in cell cycle-related genes, including cenpf, which encodes a kinetochore-related protein. Analysis of a cenpf mutant revealed impaired regeneration associated with increased levels of cardiomyocyte binucleation. Based on these findings the authors propose a model where foxm1 regulates cardiomyocyte proliferation by inducing the expression of cell cycle-related genes that ensure proper cell division.

Comments for the author

Overall, this manuscript provides some new knowledge about the molecular mechanisms that drive myocardial regeneration in zebrafish. However, several conclusions are not appropriately supported by the experimental data, and critical experiments are missing. At times, the authors overstate some of their conclusions and do not discuss limitations or alternative interpretations. Thus although this manuscript certainly shows promise, several concerns must be addressed.

Major Comments:
(1) A major concern for this reviewer is the inconsistency between the published phenotypes of foxm1 mutants in mammals and the zebrafish strain used here. In mammals, global or cardiomyocyte-specific loss of foxm1 results in perinatal lethality (PMID: 9843684, 15531365, 21779394). These studies already show a ploidy-related phenotype in cardiomyocytes in the absence of foxm1. The absence of a developmental phenotype in the foxm1sa10708 strain is, therefore concerning and may suggest that this allele is hypomorph or rescued by genetic compensation. Unfortunately, the authors never discuss this or characterize their mutant in sufficient depth (i.e., demonstrating that the mRNA levels are affected for one or all possible splicing isoforms or discussing what domains of the truncated protein are absent in this mutant). How do the authors explain this complete absence of phenotype during growth? What is the ploidy of the foxm1-/- cardiomyocytes? This is only measured in cenpf-/- (see below). Are foxm1-/- hearts completely normal or somewhat smaller or hypertrophic?

© 2023. Published by The Company of Biologists under the terms of the Creative Commons Attribution License (https://creativecommons.org/licenses/by/4.0/).

(2) The finding that foxm1-/- animals fail to regenerate should be better supported. Specifically:
* The difference in collagen accumulation in Fig. 1G-I is not convincing enough to support this claim. The authors should provide magnifications of the injured areas. To this reviewer, these three representative hearts show relatively normal regeneration for a 30 days-post amputation heart, with partial or complete recovery of the myocardium. The AFOG should be complemented with immunofluorescence with myocardial markers to assess the extent of myocardial regeneration. The same applies to Fig. S3 – these two hearts look regenerated in my opinion.
* The authors report the scar area in these animals, but it is unclear how they distinguish the scar (i.e., based on blue pixels from the AFOG staining?). Moreover, the measurement reported here does not account for differences in ventricular size. The gold standard to report scar accumulation and regeneration is to measure the % of the scar area relative to the ventricle area (see PMID: 21473762).

(3) Regarding the expression pattern of foxm1, the authors convincingly show that this gene is expressed in cardiomyocytes but not in epicardial cells. While this analysis is certainly important, this reviewer can also detect expression in endocardial cells in Fig. S1. Indeed, Foxm1 is detected in cardiomyocytes and the endocardium in mice (PMID: 21779394). While the expression in the myocardium fades postnatally, it is maintained in the endocardium and coronary vessels in adults. The authors should be, therefore, much more conservative in the way they write down their conclusions. With the evidence provided, they cannot rule out a role of foxm1 in the endocardium or endothelium during regeneration. For example, in Fig. 2D' and E', the percentage of Mef2- PCNA+ cells (endocardial?) is also reduced in the foxm1 mutant. None of this invalidates their findings but needs to be included in their discussion and conclusions.

(4) The authors connect their regeneration phenotypes to a polyploidization defect, but this needs to be better supported. While this phenotype is undoubtedly interesting, at present, the evidence is rather superficial.

Specific comments:
* It is unclear whether the authors measured cardiomyocyte ploidy, as suggested by Figure 5J, or have just quantified nucleation. The notation that they use ("1 x 2c" or "2 x 2c") implies DNA measurements per cardiomyocyte, but there are no details in the methods sections about how they did this. Claiming that all mononucleated cardiomyocytes are diploid (1 x 2c) is conceptually wrong. I suspect that they measured nucleation only, which means they might be underestimating their cardiomyocyte ploidy tremendously (i.e., if they have 4c or 8c mononucleated cardiomyocytes).
* To claim that foxm1 regulates cardiomyocyte proliferation after injury via cenpf, the authors need to perform also their ploidy quantifications in foxm1 mutants. In this version of the manuscript, they only analyze the phenotype of cenpf m/m animals, but this is insufficient to claim that the same will happen in foxm1 animals, which is their overarching hypothesis.

(5) The discussion covers many topics that are not the focus here and have been briefly mentioned in passing (i.e., metabolic changes related to pfkfb4b and pkmb). In addition, previous findings related to polyploidy in foxm1 KO mice are not discussed.

Rigor and reproducibility:
(1) The authors need to review all figures and provide details about their biological replicates and statistical tests. At present, the reporting of this information is largely inconsistent. For example, in Figure 1C. How many biological replicates have been used in the qPCR experiments described here? No information is provided in the figure legend. The materials and methods describe vagely that "a minimum of eight hearts were pooled together for each condition" and that "at least two independent biological replicates were performed". Please provide specifically the number of replicates. What is the statistical test that the authors used here?
(2) Please add scale bars in AFOG histologies (i.e., Fig. 1G-I) to ensure the size of the hearts is approximately equivalent.
(3) RNAScope is a powerful and quantitative technique. The authors could strengthen some of their conclusions by quantifying the signal in RNAScope ISHs. For example, in figure 5G-H, a reduction in prc1b is not particularly obvious and this claim would be better supported by quantifying the number of dots.

© 2023. Published by The Company of Biologists under the terms of the Creative Commons Attribution License (https://creativecommons.org/licenses/by/4.0/).

Additionally, they should compare equivalent areas in RNAScope images. For example, figure 5G and H come from what looks like two anatomically different regions (5G includes the cortical muscle, 5H looks to come mainly from the trabecular muscle; this has implications regarding the potential of different cardiomyocytes to contribute to regeneration). In addition, I do not see the injured region in Fig. 4, foxm1m/m. Difficult to compare with the rest of the genotypes.

Minor Comments:
(1) The authors need to justify why they are combining the dusp6 and WT RNASeq in Figure 1 to find foxm1 and other potential candidates. From a narrative perspective, this is just not addressed and looks unnecessary. Given that dusp6 mutants show enhanced regeneration, is foxm1 elevated in these mutants? I'm not asking to remove this experiment, but to explicitly state why they chose this strategy.

(2) One wonders whether showing the expression of foxm1 and hmmr from a single cell RNASeq from a pre-existing dataset is relevant enough to present this information in one of the central figures.

(3) "Binucleation is caused by cytokinesis failure and is a hallmark of cardiomyocyte maturation in mammals". This sentence is conceptually wrong. Please notice that binucleation is characteristic of some mammals, but not all. For example, while most cardiomyocytes in mice are binucleated (2 x 2c), this is not the case in humans. Only around 30% of the human adult cardiomyocytes are binucleated (2 x 2c, or 2 x 4c), while most are mononucleated polyploid 1 x 4c or 1 x 8c.

(4) Please also reference Windmuller 2020 when discussing the effects of deleting ect2 in cardiomyocyte polyploidization.

(5) The Mef2 antibody that the authors used in this study is not specific for Mef2c. This antibody recognizes several Mef2 proteins (including Mef2A). Please correct this in the text and figure labels.

Reviewer 3

Advance summary and potential significance to field

The manuscript by Zuppo et al aims to identify novel transcriptional regulators of cardiomyocyte proliferation in the regenerating zebrafish heart. RNA-seq following injury from wild type and Dusp6 mutants, that show accelerated regeneration, demonstrate an increase in foxm1 and hmmr levels. Specifically, foxm1 was increased in border zone cardiomyocytes in wild type mice and was detected in proximity to nrg1 positive cells following injury, while deletion of foxm1 reduces myocyte proliferation. Foxm1 mutants show a decrease in cell cycle and glucose metabolism genes. In addition, cenpf, which is a foxm1 target gene, is reduced in foxm1 mutants. Cenpf deletion impaired regeneration without reducing the number of PCNA positive cardiomyocytes, but rather through increased levels of cardiomyocyte binucleation indicating cytokinesis impairment. Thus, the authors conclude that foxm1 regulates cardiomyocyte cell cycle via its target cenpf during heart regeneration. Defining the transcriptional control of cardiomyocyte proliferation has significant potential to identify strategies to induce adult heart regeneration.

Comments for the author

Although defining the transcriptional control of cardiomyocyte proliferation has significant potential to identify strategies to induce adult heart regeneration, however; a few questions need to be addressed:

- Both foxm1 and cenpf have been demonstrated previously to regulate mammalian cardiomyocyte proliferation, thus why focusing on these candidates from the RNAseq data remains unclear.

- It is surprising that the zebrafish mutants develop normally, although embryonic deletion in mice disrupts heart development. Are they injury-responsive genes in zebrafish in contrast to the mammalian heart? Defining these roles are essential to highlight the novelty of these factors in

© 2023. Published by The Company of Biologists under the terms of the Creative Commons Attribution License (https://creativecommons.org/licenses/by/4.0/).

zebrafish heart regeneration and to further understand the distinct mutant phenotypes between zebrafish and mouse development.

- Although there are higher levels of binucleation in the cenpf mutants, most myocytes remain mononucleated, so are these levels sufficient to inhibit regeneration or are there additional mechanisms at play?

- Similarly, foxm1 deletion reduces PCNA levels but cenpf mutants only impact binucleation, so seems that cenpf is one target by which foxm1 blocks the regenerative response and additional targets probably control its role in regeneration.

- Minor comment: There is no Figure 6.

First revision

Author response to reviewers' comments

Response to reviewers' comments and concerns.

We thank the three reviewers for taking the time to read our manuscript and provide critical comments on how to improve this study. We have now addressed these concerns and questions with new experiments and included data to further support the findings in this study. Outlined below are our answers in addressing each of these points. In the revised version of the manuscript, we have used red text color to indicate the changes we have made to the original submission.

In this revision we have added the following new experiments:

1) Used AG1478 in *erbb2* heterozygous zebrafish to determine if suppressing the Nrg1 receptor will result in decreased foxm1 expression. This is now presented in Fig 3I.
2) EdU and Mef2 labelling of dividing CMs at 7, 10 and 14 dpa in cenpf mutant zebrafish to determine if CM proliferation is increased at later time points. This is presented in Figure S7.
3) We have performed quantification of RNAscope images to determine *irs1* and *prc1b* expression in foxm1 and cenpf mutants.
4) Analyzed RNAseq data from 7 and 20 dpa to determine *foxm1* and *hmmr* expression. The processed data is now included in Supplementary Table 1.

A PDF of our point-by-point response has been uploaded to the Supplementary Information.

Again, we thank the reviewers in advance for taking the time to review this revision.

Reviewer 1

In this study, Zuppo and colleagues identified the transcription factor foxm1 as a requirement for cardiomyocyte proliferation during adult zebrafish heart regeneration. By conducting RNA-seq in uninjured and 3 dpa WT and dusp6 mutant ventricles, they observed enhanced foxm1 expression after cardiac injury at 3 dpa. They also re- evaluated a published scRNA-seq dataset of embryonic mouse hearts, which demonstrated Foxm1 expression in cycling murine cardiomyocytes. foxm1m/m revealed declined cardiomyocyte proliferation at 7 dpa and defective myocardial recovery with enhanced fibrosis at 30 dpa in comparison to wild-type ventricles. Further, foxm1 was predominantly expressed in border zone cardiomyocytes and RNA-seq analysis of wild- type and foxm1m/m ventricles at 3 dpa uncovered decreased expression of cell cycle progression, insulin signaling, glycolysis, and AP-1 factors, implicating foxm1 as a regulatory factor in cardiomyocyte division. Moreover, this RNA-seq dataset identified diminished expression of cenpf in foxm1m/m ventricles in comparison to wild-type at 3 dpa. Examination of cenpf mutants demonstrated large fibrotic scars and increased cardiomyocyte binucleation at 30 dpa. Overall, the authors

© 2023. Published by The Company of Biologists under the terms of the Creative Commons Attribution License (https://creativecommons.org/licenses/by/4.0/).

demonstrate the requirement of foxm1 and cenpf for effective cardiac regeneration; this will contribute to further findings and help isolate regulatory differences in zebrafish and mammalian injury responses in cardiomyocytes that could potentially be utilized to enhance regeneration in humans.

Major Concerns/Questions

1. The authors chose 3 dpa as the key time point for early zebrafish regeneration after ventricle resection to collect hearts for RNA-seq, but do not disclose their reasoning for this time point except the description "Cardiomyocytes respond to induced stimuli at 3 days post-amputation (dpa) and begin to dedifferentiate and divide". It would be helpful to readers to include why this time point not peak time like 7 dpa was chosen.

   We thank the reviewer for raising this point. In this revision, we have now analyzed RNA-seq data for 7 and 20 dpa for WT and *dusp6* mutant hearts and will include this dataset in this study. This is included in Supplementary Table 1 showing increased and decreased gene expression at 3, 7 and 20dpa. We have also added data to show average TPM for *foxm1* and *hmmr* at 3, 7 and 20 dpa, in conjunction with the log2FC with uninjured as baseline expression (See Supplementary Figure S1). As the reviewer will note expression of *foxm1* and *hmmr* were highest at 3 dpa but was still increased at 7dpa. However, both genes were expressed at similar levels to uninjured hearts at 20dpa.

2. The combination of the RNA-seq data from both WT and dusp6 mutant hearts is not explained. Was there no difference in DGE between WT and dusp6 mutant hearts at the time points observed (no injury and 3 dpa)?

   These were single experiments at these time points for each condition. Since we were looking for gene expression changes in injured vs uninjured we combine these data sets, with the knowledge that *dusp6* mutant hearts regenerated at an accelerated rate. While our intention was to determine if *dusp6* mutants showed further increased expression of key regenerative genes, it was naïve on our part to think that a single bulk RNA-seq experiment would identify these. One factor at the time of doing these experiments was limited by resources at that time. We followed up on candidate genes by multiple qPCR experiments and with fluorescent in situ hybridization (RNAscope) studies.

3. In results subsection "Identification of mitotic genes involved in cardiac regeneration section" paragraph 1, the use of cluster in the phrase "Many genes identified in this cluster" is somewhat confusing. I infer that the authors are referring to the genes that were significantly enhanced after cardiac injury at 3 dpa, and suggest rephrasing to make the sentence clearer.

   We have changed the term "cluster" to gene category based on NIH DAVID GO terms. The sentence now reads: "Many increased expression genes at 3dpa include *ect2, plk1,* and *hmmr* [46-48], are known to play critical roles in zebrafish cardiac regeneration and detecting these transcripts demonstrated that these injured hearts were in a proliferative state (**Supplementary Table 1**).

4. In qPCR to validate RNA-seq data in Results "Identification of mitotic genes involved in cardiac regeneration section", are WT and dusp6 mutant hearts combined as with RNA-seq or are just WT hearts used to confirm RNA-seq results? Further, as hmmr is selected as an interesting candidate, hmmr gene expression should also be included in the representative qPCR in Fig. 1C.

   Only cDNA from WT uninjured and WT 3dpa hearts was used for the qPCR validation experiments in Figure 1. *hmmr* qPCR was not performed in this instance as a previous published study already documented this from our group. Please see Missinato et al. Cardiovasc Res. 2015 Sep 1;107(4):487-98. doi: 10.1093/cvr/cvv190. Here we used a proteomics approach to identify differential protein expression in hearts at 7dpa vs

© 2023. Published by The Company of Biologists under the terms of the Creative Commons Attribution License (https://creativecommons.org/licenses/by/4.0/).

uninjured. In the Missinato study, a *hmmr* mutant was not available at that time so we used this opportunity to explore its function in heart regeneration.

5. In the supplementary Figure 1 A-D, the authors need to include the images of individual myl7:GFP or wt1b:GFP fluorescence and foxm1 fluorescence in addition to the image showing both signals. It is hard to see foxm1 red fluorescence at 3 and 7 dpa due to strong myl7+ green signals. Additionally, it appears as though 3 dpa foxm1 expression in the wt1b:GFP/foxm1 image is strongly expressed throughout the myocardium and not just in border zone cardiomyocytes, is this due to overexposure or high signal background for in situ foxm1 signals that are very different from myl7:EGFP/foxm1 images?

    We have now included images of individual myl7:EGFP and wt1b:EGFP cells and split the channels showing *foxm1* expression in Supplementary Figure S3. We agree that there was high background and overexposure in the wt1b:EGFP experiment as a means to certain that there is no *foxm1+* staining in the epicardial lineages.

6. Does foxm1m/m influence myocardial regeneration at 30 dpa? Although the authors observed a decline during the peak time point of CM proliferation at 7 dpa, it would be necessary to compare myocardial recovery in wild-type and foxm1m/m at 30 dpa as the direct evidence to link the requirement of foxm1 for cardiomyocyte proliferation.

    foxm1 expression seems to decrease after 7dpa and is almost absent at 20dpa based on the bulk RNAseq data (see Supplementary Figure S1). Based on these observations we conclude that *foxm1* is induced from 3dpa, remains expressed at 7dpa but by 20dpa, expression has return to baseline. The expression profile of *foxm1* mirrors that of cardiomyocyte proliferation as reported by Poss et. al. Science. 2002 Dec 13;298(5601):2188-90.

7. which cell types are these nrg1+ cells observed in Figure 3A and B, are from epicardial-derived cells? Other cell types, like Treg cells, also express nrg1 after cardiac injury in zebrafish (Hui et al., 2017 PMID: 29257949).

    We like to thank the reviewer for raising the point that *nrg1* is expressed in *foxp3* expressing Treg cells and these have been reported to infiltrate the injured hearts. We did not determine the source of *nrg1+* cells but agree that both perivascular and Tregs *nrg1+* cells could stimulate neighboring cardiomyocytes to express *foxm1*. We have included text to cite the study by Hui et. al.

8. For supplementary Figure 2 A and B, there appears to be little to no difference in the representative figures shown between WT and foxm1m/m. The authors need to measure the signal intensity for irs1 signals at 3 dpa in the border region in WT and foxm1m/m. Also, it would be useful to include lower magnification images of the hearts, to better understand where foxm1 is expressed.

    As suggested, we have now quantified the *irs1* signals and report significant lower expression in *foxm1* mutant hearts. This is in line with the bulk RNAseq and the qPCR experiments. The quantification is now included in Figure S4E.

9. Can the authors include the data to show foxm1+ cardiomyocytes are proliferating cells by combine foxm1 in situ hybridization and MEF2/PCNA staining or EdU incorporation?

    We have attempted to stain with anti-PCNA antibody and combined with *foxm1* RNAscope in situ hybridization. Unfortunately, we were not able to detect the PCNA staining, and this may be due to the RNAscope target retrieval procedure, which requires heating the tissue sections to 90-98ºC for 3 minutes. However, we have performed RNAscope with three probes (*cenpf*, *g2e3* and *foxm1*) and show that these are co-expressed in cardiomyocytes that are close to the border zone (see Figure 4 and new supplementary figure S5).

© 2023. Published by The Company of Biologists under the terms of the Creative Commons Attribution License (https://creativecommons.org/licenses/by/4.0/).

10. Figure 4D: it is very hard to see the triple positive signals for in situ foxm1, cenpf, and g2e3. There appears to be high green background from cenpf fluorescence signals, and the yellow color for g2e3 is difficult to observe. A different color will be used to represent g2e3 in Figure 4D. Also, it would be beneficial to include here or in the supplement, images of each in situ signal and then the image of all 3 signals together with DAPI. Additionally, the placement of the white arrowheads for the “triple positive” cells is5omewhatt ambiguous, they need to be placed closer to cells. It looks like most of the white arrowheads for foxm1m/m image at 3 dpa and 7 dpa are not labelling a “triple positive” cell, I do not see yellow signals for g2e3 in the bottom left cell labelled by arrowhead at 3 dpa or in any of the cells labelled by arrowhead at 7 dpa. It would be better to use a different color to label example cells that are not “triple positive” in foxm1m/m and use white coloring for “triple positive” cells and explain what arrowheads/colors are labeling in the figure legend.

    The zebrafish heart does have autofluorescence in the 488/fluorescein channels due to the presence of cardiolipins and this autofluorescence is also present (in a reduced extent) in the Cy3 channel. With the fluorescent labeling kit provided by ACD Bio for RNAscope, we were limited to the secondary dyes of fluorescein, Cy3, and Cy5. Hence, one probe of the 3 in each data set will have some level of background. We did not significantly reduce the background of any probes detected with fluorescein dye as we did not wish to artificial modify the image. We do agree that the yellow color is difficult to detect. To show the co-expression of these genes, we have split the image into distinct color channels (foxm1: red, g2e3: yellow and cenpf: green) and highlight the cardiomyocytes with co-expression. We have included this as a new supplementary figure S5.

11. The authors proposed that foxm1 drives the expression of cell cycle genes and promote cardiomyocyte proliferation. They showed g2-phase gene ccng2 is decreased in foxm1 mutant in Fig.4C. However, ccng2 was reported to negatively regulate cell proliferation (Zimmermann et al., Cell Cycle 2016). The authors need to explain or discuss.

    We thank the reviewer in raising the question on the role of Ccng2 in proliferation. There are numerous studies that show the inverse correlation of Ccng2 expression and proliferation of several cell types. While our data shows overall decrease in ccng2 in *foxm1* mutants, we did not specifically address *ccng2* expression in cardiomyocytes. Thus, we are not able to definitively state its role. We agree that our statement is misleading and have not included mentioning this RNAseq data in the text. This *ccng2* data is listed in the dataset in Supplementary Table 3. We believe studies that directly address ccng2 is beyond the scope of this study.

12. The authors showed loss of foxm1 decreases cardiomyocyte proliferation and enlarged scar deposition. As foxm1 is proposed to regulate cardiomyocyte cycling, the authors need to explain or discuss why loss of foxm1 impairs fibrotic resolution?

    Since *foxm1* impairs cell proliferation in general, there is the possibility that other cell types are also affected by global gene mutation, including fibroblasts and M2 macrophages. There is evidence that loss of Foxm1 prevents fibrotic resolution as the macrophage-specific deletion of *Foxm1* caused increased expression of pro-inflammatory cytokines in pulmonary macrophages (Goda et al., Plos Genetics PMID: 32271749), so it could be that this is also present in macrophages responding to the ventricular injury in our zebrafish. The increase of pro-inflammatory cytokines could prolong fibrosis by preventing the removal of fibrin and collagens due to reduced level of the pro-regenerative macrophages. We have added several sentences on this possible role to the discussion but performing experiments to test this hypothesis goes beyond the scope of this project.

13. Figure 5G,H. Quantification of the myocardium prc1b in situ signals in WT and cenpfm/m hearts would be beneficial, and lower magnification images of the injured hearts will be included. The representative image of prc1b signals in cenpfm/m ventricles should show

© 2023. Published by The Company of Biologists under the terms of the Creative Commons Attribution License (https://creativecommons.org/licenses/by/4.0/).

less of the injury site and more of the border zone myocardium like in the WT figure in panel G for a better comparison between the wild-type and mutant ventricles.

> We quantified the both the number of *prc1b* probe within the myocardium and *prc1b* probe fluorescent intensity from multiple regions of interest, but we detected no significant decrease in *prc1b* expression. Based on this, we have amended this section to reflect that there is no change in *prc1b* expression while keeping the images to depict the lack of change in a qualitative manner. This is now included in Supplementary Figure S6.

Minor Concerns/Comments

1. In the introduction, pg. 1 paragraph 2: insert "for" between required and ATP to update sentence as follows: "Mature cardiomyocytes use fatty acid oxidation to generate substrates required for ATP generation via oxidative phosphorylation"

   Added "for"

2. In the introduction, pg. 2 paragraph 3: provide full name of AP-1 (activator protein-1) before using the abbreviated name

   Added "activator protein-1"

3. Figure 2: Please provide name of the myocardium reporter used to the left of Fig.2 A and A', is it myl7:GFP? Please add for clarity in figure 2 and add in the figure 2 legend.
   - In figure 2A and A', the myocardium fluorescence is from autofluorescence at 488nm. We have stated this in the figure legend as: "The myocardium is marked by 488nm as background fluorescence."

4. Figure 2 Legend does not match with the Figure names in Figure 2 and the text of the manuscript: WT is labeled as D-E but should be D-D' and foxm1m/m is labelled as F-G but should be E-E', label H should be changed to F. There is also an empty box symbol before 0.0001 p-value in figure legend, this should be removed or the appropriate symbol inserted/fixed.
   This have now been fixed to match the figure 2 legend to the figure.

5. Supplementary Figure S1 Legend: What do the white and yellow arrowheads label?
   This was a mistake. There should be only one color arrowheads to signify *foxm1* probe labelling.

6. Figure 3A: Add a box to indicate the region of the ventricle highlighted in Figure 3B
   A white box is added to Figure 3A as suggested.

7. Figure 5 Legend: There is a box symbol before 0.0001 in description of p-values, should be removed/fixed
   This must be the probably generated by conversion to pdf. We will check the pdf version of the manuscript.

8. Figure 5I: May be helpful for readers if panels are split into 2 sections with example images of mononucleated and binucleated cardiomyocytes separated and labeled.
   We have split the panels to indicate bi- and mono-nucleated cardiomyocytes and used yellow arrowheads to mark the nuclei in the cells.

9. Figure 6 legend: There is no Figure 6, this legend/info should be removed.
   Figure 6 is now included. We apologize for its absence.

10. Supplementary Figure S2 legend: The labeling throughout the legend is off and does not match the manuscript text and Supp. Fig. S2. There are no uninjured hearts shown in the figure so mention of these in the legend should be removed. Needs to be changed so that

© 2023. Published by The Company of Biologists under the terms of the Creative Commons Attribution License (https://creativecommons.org/licenses/by/4.0/).

(A) is 3 dpa WT hearts with irs1, (B) is 3 dpa foxm1m/m with irs1, (C) is WT 3 dpa hearts with prc1b and (D) is 3 dpa foxm1m/m with prc1b.

We have now corrected this figure legend.

Reviewer 2

Overall, this manuscript provides some new knowledge about the molecular mechanisms that drive myocardial regeneration in zebrafish. However, several conclusions are not appropriately supported by the experimental data, and critical experiments are missing. At times, the authors overstate some of their conclusions and do not discuss limitations or alternative interpretations. Thus, although this manuscript certainly shows promise, several concerns must be addressed.

<u>Major Concerns/Questions</u>

1. A major concern for this reviewer is the inconsistency between the published phenotypes of foxm1 mutants in mammals and the zebrafish strain used here. In mammals, global or cardiomyocyte-specific loss of foxm1 results in perinatal lethality (PMID: 9843684, 15531365, 21779394). These studies already show a ploidy-related phenotype in cardiomyocytes in the absence of foxm1. The absence of a developmental phenotype in the foxm1sa10708 strain is, therefore concerning and may suggest that this allele is hypomorph or rescued by genetic compensation. Unfortunately, the authors never discuss this or characterize their mutant in sufficient depth (i.e., demonstrating that the mRNA levels are affected for one or all possible splicing isoforms or discussing what domains of the truncated protein are absent in this mutant). How do the authors explain this complete absence of phenotype during growth? What is the ploidy of the foxm1-/- cardiomyocytes? This is only measured in cenpf-/- (see below). Are foxm1-/- hearts completely normal or somewhat smaller or hypertrophic?

   We are also surprised that the loss of *foxm1* does not result in an embryonic phenotype as has been reported in mice. We did observe that the male *foxm1* homozygous mutants are infertile and have never been able to successfully fertilize eggs suggesting that *foxm1* mutants do have a fertility issue in males. The *foxm1* ENU-induced mutation from the Sanger Consortium is predicted to be within the forkhead DNA binding domain. Thus, we believe that the mutation is damaging and it is not likely a splice variant can compensate for the forkhead DNA binding function. One reason for the lack of embryonic phenotype is that there exist many related Fox genes. Many have been documented to play a role in proliferation either positively or negatively. In zebrafish this is further complicated with the duplication of the genome and therefore compensation is often observed when genes are mutated as previously reported. We believe that there are other forkhead genes that compensate for the loss of *foxm1* in the zebrafish. For example, Foxk1 and Foxk2 have been shown to be important for regulating proliferation and these are expressed in the embryo (from ZFIN). A detailed analyses of compensation of Fox genes in development is beyond the scope of this study.

   With regards to *foxm1* RNA expression in the *foxm1 sa10708* line, we did not detect expression at 3dpa as was observed in WT hearts (see Figure 4D). This suggests that either mutant *foxm1* transcript is degraded by non-sense mediated decay or that expression of *foxm1* is regulated by Foxm1 protein.

   In new data, we present ploidy counts of cardiomyocytes at 20dpa in Figure 5H. We did not observe increased binucleated CMs in the *foxm1* mutant hearts. One reason for this is that cardiomyocyte proliferation is significantly reduced in the *foxm1* mutant hearts and thus binucleation will not be different from WT if there are much fewer cells entering the cell cycle.

2. (2) The finding that foxm1-/- animals fail to regenerate should be better supported. Specifically:
   * The difference in collagen accumulation in Fig. 1G-I is not convincing enough to

© 2023. Published by The Company of Biologists under the terms of the Creative Commons Attribution License (https://creativecommons.org/licenses/by/4.0/).

support this claim. The authors should provide magnifications of the injured areas. To this reviewer, these three representative hearts show relatively normal regeneration for a 30 days-post amputation heart, with partial or complete recovery of the myocardium. The AFOG should be complemented with immunofluorescence with myocardial markers to assess the extent of myocardial regeneration. The same applies to Fig. S3 – these two hearts look regenerated, in my opinion.

* The authors report the scar area in these animals, but it is unclear how they distinguish the scar (i.e., based on blue pixels from the AFOG staining?). Moreover, the measurement reported here does not account for differences in ventricular size. The gold standard to report scar accumulation and regeneration is to measure the % of the scar area relative to the ventricle area (see PMID:21473762).

We thank the reviewer for pointing this out. We have now used two different approaches to score for the resolution of scar tissue in ventricular resected hearts. We used the method as suggested by the reviewer to quantify % of scar area. This is now included in Supplementary Figure S2 and S6. We also measure by semi-quantitative scoring of scar tissue and use Fisher's Exact test to determine statistical significance. This in now included in Figure 1J and Supplementary Figure S6. In addition, we have now included representative images of AFOG staining at 30dpa in Supplementary Figure S2 to show more representative images obtained for WT, *foxm1* and *hmmr* mutant hearts.

3. Regarding the expression pattern of foxm1, the authors convincingly show that this gene is expressed in cardiomyocytes but not in epicardial cells. While this analysis is certainly important, this reviewer can also detect expression in endocardial cells in Fig. S1. Indeed, Foxm1 is detected in cardiomyocytes and the endocardium in mice (PMID: 21779394). While the expression in the myocardium fades postnatally, it is maintained in the endocardium and coronary vessels in adults. **The authors should be, therefore, much more conservative in the way they write down their conclusions.** With the evidence provided, they cannot rule out a role of foxm1 in the endocardium or endothelium during regeneration. For example, in Fig. 2D' and E', the percentage of Mef2- PCNA+ cells (endocardial?) is also reduced in the foxm1 mutant. None of this invalidates their findings but needs to be included in their discussion and conclusions.

We agree with the reviewer's comment on being more conservative and we have amended this section in the paper to reflect this. We will add statements that state that Foxm1 has been shown to be expressed in the endocardium in mice and that there is a possibility that Foxm1 may function in the endocardium during regeneration. The text included as follows:

"This does not exclude the possibility that *foxm1* transcript is not expressed in other cells residing in the myocardium, such as endocardial cells, but this data suggests that *foxm1* expression is predominantly localized to border zone cardiomyocytes after cardiac injury."

With respect to Mef2+/PCNA+, this population is considered cardiomyocytes in the adult zebrafish heart. While we have not excluded the possibility that the loss of proliferation is also in endocardial cells, we have included text to state this as follows: "While *foxm1* is also expressed in other cell types during development including the endocardium, we have not ruled out proliferation deficient in this population."

4. The authors connect their regeneration phenotypes to a polyploidization defect, but this needs to be better supported. While this phenotype is undoubtedly interesting, at present, the evidence is rather superficial.

Specific comments:

* It is unclear whether the authors measured cardiomyocyte ploidy, as suggested by Figure 5J, or have just quantified nucleation. The notation that they use ("1 x 2c" or "2 x 2c") implies DNA measurements per cardiomyocyte, but there are no details in the methods sections about how they did this. Claiming that all mononucleated cardiomyocytes are diploid (1 x 2c) is conceptually wrong.

© 2023. Published by The Company of Biologists under the terms of the Creative Commons Attribution License (https://creativecommons.org/licenses/by/4.0/).

I suspect that they measured nucleation only, which means they might be underestimating their cardiomyocyte ploidy tremendously (i.e., if they have 4c or 8c mononucleated cardiomyocytes). We can change our phrasing here. We only checked the nucleation (mononucleated vs binucleated CMs) of these different conditions as we had difficulty measuring the actual cell ploidy of zebrafish cardiomyocytes. There were challenges using FACS cardiomyocytes from adult zebrafish hearts such that they can be labelled as mono or multinucleated cells. Therefore, there is still the possibility that the mononucleated CMs present in both the foxm1 and cenpf mutants may contain a whole range of ploidy values. We will state that other ploidy states can exist but at least bi-nucleation was quantified in these experiments in cenpf and foxm1 mutant hearts. We have added the text to state this as follows: “In contrast, no increase of binucleated cardiomyocytes in *foxm1*$^{m/m}$ was detected, which we reason to be due to the lack of cell-cycle activity in the mutant hearts, but this does not exclude the possibility of polyploidy as this phenomenon was reported in both cardiomyocytes and hepatocytes from *Foxm1* KO mice (Korver et al., 1998; Krupczak-Hollis et al., 2004; Ramakrishna et al., 2007).”

* To claim that foxm1 regulates cardiomyocyte proliferation after injury via cenpf, the authors need to perform also their ploidy quantifications in foxm1 mutants. In this version of the manuscript, they only analyze the phenotype of cenpf m/m animals, but this is insufficient to claim that the same will happen in foxm1 animals, which is their overarching hypothesis.

As addressed in this reviewer’s previous concern (Major Concern #1), we have included ploidy counts of *foxm1*$^{m/m}$ cardiomyocytes at 20dpa in Figure 5H and we detected no decrease in bi-nucleation compared to WT 20dpa cardiomyocytes. While we were unable to extend these studies to polyploidization we have included test to state this limitation. See above point.

5. The discussion covers many topics that are not the focus here and have been briefly mentioned in passing (i.e., metabolic changes related to pfkfb4b and pkmb). In addition, previous findings related to polyploidy in foxm1 KO mice are not discussed.

   We have made large changes to the Discussion section where we refocus on the major topics of this study. We have also included mention on the possibility of changes in polyploidy in the adult zebrafish cardiomyocyte based on previous findings in foxm1 KO mice that we were unable to pursue in this work.

Rigor and Reproducibility

1. The authors need to review all figures and provide details about their biological replicates and statistical tests. At present, the reporting of this information is largely inconsistent. For example, in Figure 1C. How many biological replicates have been used in the qPCR experiments described here? No information is provided in the figure legend. The materials and methods describe vagely that "a minimum of eight hearts were pooled together for each condition"and that "at least two independent biological replicates were performed". Please provide specifically the number of replicates. What is the statistical test that the authors used here?

   We thank the reviewers for catching this. We have now included in the methods section our qPCR experiments. For each biological experiment, 8 hearts were used to isolate total RNA per condition. At least 2 biological replicates were conducted. The qPCR was only one approach to validate the RNAseq experiment and we further tested this with RNAscope for the genes that were further studied. Since these were only two biological replicates in some cases, statistical analyses were not applied.

   Please add scale bars in AFOG histologies (i.e., Fig. 1G-I) to ensure the size of the hearts is approximately equivalent.

   We have added 50µm scale bar to Figure 1G and all AFOG stained hearts.

2. RNAScope is a powerful and quantitative technique. The authors could strengthen some of their conclusions by quantifying the signal in RNAScope ISHs. For example, in figure 5G-H, a

© 2023. Published by The Company of Biologists under the terms of the Creative Commons Attribution License (https://creativecommons.org/licenses/by/4.0/).

reduction in prc1b is not particularly obvious, and this claim would be better supported by quantifying the number of dots. Additionally, they should compare equivalent areas in RNAScope images. For example, figure 5G and H come from what looks like two anatomically different regions (5G includes the cortical muscle, 5H looks to come mainly from the trabecular muscle; this has implications regarding the potential of different cardiomyocytes to contribute to regeneration). In addition, I do not see the injured region in Fig. 4, foxm1m/m. Difficult to compare with the rest of the genotypes.

As pointed out by reviewer 1, we have now quantified the fluorescent intensity of the RNAscope probes or counted the number of probes in between WT and mutant hearts. Using this approach, we were able to measure average expression of RNAscope probes and performed Students t-test. This is now shown in Figure S4 and S6.

Minor Comments

1. The authors need to justify why they are combining the dusp6 and WT RNASeq in Figure 1 to find foxm1 and other potential candidates. From a narrative perspective, this is just not addressed and looks unnecessary. Given that dusp6 mutants show enhanced regeneration, is foxm1 elevated in these mutants? I'm not asking to remove this experiment, but to explicitly state why they chose this strategy.

   This was also a concern from Reviewer 1. Our initial goal was to determine if there were differences between dusp6 mutant and WT hearts using bulk RNA seq. Since this was our first attempt and resources were limited at the time, such difference was not observed in the RNA seq data. We combined these RNAseq experiments and classified as injured vs uninjured hearts to identify genes involved in regeneration.

2. One wonders whether showing the expression of foxm1 and hmmr from a single cell RNASeq from a pre-existing dataset is relevant enough to present this information in one of the central figures.

   This is relevant as it shows that Foxm1 is expressed in proliferating cardiomyocytes, particularly in G2/M phase of the cell cycle. This suggests that foxm1 is required for normal embryonic CM proliferation and in the context of heart regeneration in the zebrafish, foxm1 is activated for proliferation.

3. " Binucleation is caused by cytokinetic failure and it is theorized that these binucleated cardiomyocytes fail to re-enter the cell cycle following cardiac injury (Hesse et al., 2018; Leone et al., 2018; Liu et al., 2019). ". This sentence is conceptually wrong. Please notice that binucleation is characteristic of some mammals, but not all. For example, while most cardiomyocytes in mice are binucleated (2 x 2c), this is not the case in humans. Only around 30% of the human adult cardiomyocytes are binucleated (2 x 2c, or 2 x 4c), while most are mononucleated polyploid 1 x 4c or 1 x 8c.

   We thank the reviewer for raising this mistake. We have now corrected this sentence. The sentence now reads: “Binucleation is caused by cytokinetic failure and it is theorized that these binucleated cardiomyocytes fail to re-enter the cell cycle following cardiac injury (Hesse et al., 2018; Leone et al., 2018; Liu et al., 2019).”

4. Please also reference Windmuller 2020 when discussing the effects of deleting ect2 in cardiomyocyte polyploidization.

   This citation is now included.

5. The Mef2 antibody that the authors used in this study is not specific for Mef2c. This antibody recognizes several Mef2 proteins (including Mef2A). Please correct this in the text and figure labels.

   This is now corrected in the figures and text.

© 2023. Published by The Company of Biologists under the terms of the Creative Commons Attribution License (https://creativecommons.org/licenses/by/4.0/).

Reviewer 3

The manuscript by Zuppo et al aims to identify novel transcriptional regulators of cardiomyocyte proliferation in the regenerating zebrafish heart. RNA-seq following injury from wild type and Dusp6 mutants, that show accelerated regeneration, demonstrate an increase in foxm1 and hmmr levels. Specifically, foxm1 was increased in border zone cardiomyocytes in wild type mice and was detected in proximity to nrg1 positive cells following injury, while deletion of foxm1 reduces myocyte proliferation. Foxm1 mutants show a decrease in cell cycle and glucose metabolism genes. In addition, cenpf, which is a foxm1 target gene, is reduced in foxm1 mutants. Cenpf deletion impaired regeneration without reducing the number of PCNA positive cardiomyocytes, but rather through increased levels of cardiomyocyte binucleation indicating cytokinesis impairment. Thus, the authors conclude that foxm1 regulates cardiomyocyte cell cycle via its target cenpf during heart regeneration. Defining the transcriptional control of cardiomyocyte proliferation has significant potential to identify strategies to induce adult heart regeneration.

Major Concerns/Comments

1. Both foxm1 and cenpf have been demonstrated previously to regulate mammalian cardiomyocyte proliferation, thus why focusing on these candidates from the RNAseq data remains unclear.

   These are true statements, but what many of these previous mammalian studies have not shown was how *Foxm1* and *Cenpf* respond in a regenerative injury setting. Some recent studies have shown that overexpression of *Foxm1* with other mitogenic factors can enhance CM proliferation in uninjured, P7 or older mice and in 8-week old mice post-MI (Cheng et al., EMBO Medicine, 2017) and that treating hearts with valproic acid post-MI increased *Foxm1* expression and was cardioprotective by preventing fibrotic progression (Tian et al., , 2020). But these models focused on non-regenerative models; our model is a model of cardiac regeneration and highlights the importance of these genes in this process.

2. It is surprising that the zebrafish mutants develop normally, although embryonic deletion in mice disrupts heart development. Are they injury-responsive genes in zebrafish in contrast to the mammalian heart? Defining these roles are essential to highlight the novelty of these factors in zebrafish heart regeneration and to further understand the distinct mutant phenotypes between zebrafish and mouse development.

   This is a similar comment from Reviewer 1 on the lack of an embryonic phenotype with foxm1 mutants. We believe that there are other forkhead genes that compensate for the loss of foxm1 in the zebrafish. Foxk1 and Foxk2 have been shown to be important for regulating proliferation and these are expressed in the embryo are just two examples. A detailed analyses of compensation of fox genes in development is beyond the scope of this study.

3. Although there are higher levels of binucleation in the cenpf mutants, most myocytes remain mononucleated, so are these levels sufficient to inhibit regeneration or are there additional mechanisms at play?

   A new experiment was conducted to address whether CM proliferation was extended in the *cenpf* mutant hearts. We did not observe a significantly increased CM proliferation index at 10-14dpf. Thus there does not appear to be a continual burst of CM proliferation at later time points and may point to a limited time window as to when CMs can divide. We tied to perform a proliferative history on CM proliferation, but we ran into technical difficulties in pursuing this.

   Similarly, foxm1 deletion reduces PCNA levels but cenpf mutants only impact binucleation, so seems that cenpf is one target by which foxm1 blocks the regenerative response and additional targets probably control its role in regeneration.

   This is true, as we were able to show that other cell cycle genes such as prc1b and g2e3 were also decreased in the foxm1 mutant, but not in the cenpf mutant hearts. We have not

© 2023. Published by The Company of Biologists under the terms of the Creative Commons Attribution License (https://creativecommons.org/licenses/by/4.0/).

exhaustively look at all the potential cell cycle genes that are not activated in the *foxm1* mutant but these could be future studies.

Minor Comment

1. No Figure 6

We apologize for its absence. It is now included.

Second decision letter

MS ID#: DEVELOP/2022/201163

MS TITLE: Foxm1 regulates cardiomyocyte proliferation in adult zebrafish after cardiac injury

AUTHORS: Daniel A Zuppo, Maria Azzurra Missinato, Lucas Santana-Santos, Guang Li, Panagiotis Benos, and Michael Tsang

I have now received all the referees reports on the above manuscript, and have reached a decision. The referees' comments are appended below, or you can access them online: please go to BenchPressand click on the 'Manuscripts with Decisions' queue in the Author Area.

The overall evaluation is positive and we would like to publish a revised manuscript in Development, provided that the referees' comments can be satisfactorily addressed. You will notice that Reviewers 1 and 3 are satisfied with the revisions, whereas Reviewer 2 expresses remaining concerns. I feel that Reviewer 2 comments are helpful and indicate a careful reading and concern for data quality, and so I have thought about some minor revisions that would help the work. If you are able to address these, which are unlikely to require further experiments, I feel this work can move forward without an additional round of review.

- Reviewer 2, comments 1 and 2: For the fibrosis assay, in Methods indicate that scoring was performed by an experimenter blinded to the genotype of the animal. Also indicate that a semiquantitative approach can be preferable to measuring collagen in such small, variable resection injuries. When described in the Results, indicate that injury sites were graded as more severe when assessed semi-qualitatively as in previous publications by other groups (citations). You might also mention that AFOG stains can lead to tissue shrinkage, which can change muscle morphology and mask typical features of regenerated muscle.

- Comment 3: I agree with the reviewer that it is general practice to incorporate the responses into the manuscript, as the reviewer’s goal is to help improve the paper for the field. Please incorporate your responses where you haven't done so at some point in the test, and indicate in highlights for the next revision.

- Comments 4 and 5: Regarding Fig. S6 and the cenf mutant, please rephrase the subtitle as it is too strong for the data (“fail to regenerate”• and “due to increased binucleation”• seem to overstate or are speculative). Per the reviewer point, I agree that the heart in S6B appears to have regenerated within normal range. Given the reviewer concerns, you should show representative images of sections from all animals as in Fig. S2. Also recommended to generally soften the language including modifying the conclusion “Therefore, the loss of cenpf caused mitotic dysregulation during heart regeneration, and because fewer cardiomyocytes divided, fibrotic scar tissue persisted”•, as this seems speculative.

- Comment 6: Similarly, to address the comment, the authors should use more cautious language throughout and indicate in Figure 6 that this is a “preliminary model”•, using the Discussion to discuss limitations, other possibilities, or necessary experiments to address this.

© 2023. Published by The Company of Biologists under the terms of the Creative Commons Attribution License (https://creativecommons.org/licenses/by/4.0/).

Please attend to all of the reviewers' comments in your revised manuscript and detail them in your point-by-point response. If you do not agree with any of their criticisms or suggestions explain clearly why this is so. If it would be helpful, you are welcome to contact us to discuss your revision in greater detail. Please send us a point-by-point response indicating your plans for addressing the referee's comments, and we will look over this and provide further guidance.

Reviewer 1

Advance summary and potential significance to field

In this study, Zuppo and colleagues identified the transcription factor foxm1 as a requirement for cardiomyocyte proliferation during adult zebrafish heart regeneration. By conducting RNA-seq in uninjured and 3 dpa WT and dusp6 mutant ventricles, they observed enhanced foxm1 expression after cardiac injury at 3 dpa. They also re-evaluated a published scRNA-seq dataset of embryonic mouse hearts, which demonstrated Foxm1 expression in cycling murine cardiomyocytes. foxm1m/m revealed declined cardiomyocyte proliferation at 7 dpa and defective myocardial recovery with enhanced fibrosis at 30 dpa in comparison to wild-type ventricles. Further, foxm1 was predominantly expressed in border zone cardiomyocytes and RNA-seq analysis of wild-type and foxm1m/m ventricles at 3 dpa uncovered decreased expression of cell cycle progression, insulin signaling, glycolysis, and AP-1 factors, implicating foxm1 as a regulatory factor in cardiomyocyte division. Moreover, this RNA-seq dataset identified diminished expression of cenpf in foxm1m/m ventricles in comparison to wild-type at 3 dpa. Examination of cenpf mutants demonstrated large fibrotic scars and increased cardiomyocyte binucleation at 30 dpa. Overall, the authors demonstrate the requirement of foxm1 and cenpf for effective cardiac regeneration; this will contribute to further findings and help isolate regulatory differences in zebrafish and mammalian injury responses in cardiomyocytes that could potentially be utilized to enhance regeneration in humans.

Comments for the author

I am satisfied with the revised version and no more comments.

Reviewer 2

Advance summary and potential significance to field

This manuscript is a reviewed version of a previous submission from Zuppo and colleagues. Overall, the authors have been only minimally responsive to my comments. Here are the specifics:

Comments for the author

This manuscript is a reviewed version of a previous submission from Zuppo and colleagues. Overall, the authors have been only minimally responsive to my comments. Here are the specifics:

* The defect at 30 dpa in the foxm1 mutant remains rather unconvincing. The authors have not addressed my comment #2 regarding providing magnifications of the injured areas and complementing the AFOGs with some immunostainings to detect myocardial recovery - this foundational element of their paper should be much better supported. In 5 out of the 7 WT hearts shown in Fig. S2, I cannot see any of the stereotypical morphology of the regenerated myocardium after resection (expansion of the cortical myocardium, etc. - see Poss et al., 2002), so I'm not convinced the authors are comparing the same areas across genotypes.

* The methods used to quantify the scar tissue remain vague. How do the authors define the scar (see comment #2 in my previous review)? Blue pixels based on AFOG? Are they arbitrarily selecting an area manually? While measuring 60 sections per heart (PMID: 21473762) is excessive, measuring just four is insufficient.

© 2023. Published by The Company of Biologists under the terms of the Creative Commons Attribution License (https://creativecommons.org/licenses/by/4.0/).

* The authors need to incorporate some of the critical explanations the three reviewers have requested into their text. While I appreciate the responses in the “Answer to reviewers”, they should include many of these pieces of information in the main text. If the reviewers asked for them, readers would likely have the same questions. For example, why are they not including the relevant information re- the difference in phenotypes between mammalian and zebrafish foxm1 mutants? (comment #1). The combined use of RNASeq from different genotypes (pointed out by reviewer 1) is also not justified in the text, just in the letter.

* The data presented in Sup Fig 6 re- the cenpf phenotype is, again, not convincing. In my opinion, the mutant heart looks regenerated. I cannot see the injured/regenerated area in the WT hearts.

* The lack of binucleation in foxm1 mutants is justified by the “lack of cell cycle activity”, but this is an overstatement. Yes, there is significantly less cardiomyocyte proliferation (from around 20% to 10%), but if their hypothesis is true, there should be some increase in binucleation in these hearts.

* I do not feel the authors have enough data to support the model from Fig. 6.
As I read this drawing, they’re proposing that loss of foxm1 results in regeneration failure involving G2/M checkpoint failure. If true, then cells would be 4n, but they have not demonstrated an increase in cardiomyocyte ploidy after injury.

Reviewer 3

Advance summary and potential significance to field

This is a revised manuscript by Zuppo et al aims to identify the role of Foxm1 on zebrafish cardiomyocyte proliferation and regeneration. The authors have addressed my questions and I have no further comments.

Comments for the author

The authors have addressed my questions and I have no further comments.

Second revision

Author response to reviewers' comments

Reviewer 1 Advance Summary and Potential Significance to Field:
In this study, Zuppo and colleagues identified the transcription factor foxm1 as a requirement for cardiomyocyte proliferation during adult zebrafish heart regeneration. By conducting RNA-seq in uninjured and 3 dpa WT and dusp6 mutant ventricles, they observed enhanced foxm1 expression after cardiac injury at 3 dpa. They also re-evaluated a published scRNA-seq dataset of embryonic mouse hearts, which demonstrated Foxm1 expression in cycling murine cardiomyocytes. foxm1m/m revealed declined cardiomyocyte proliferation at 7 dpa and defective myocardial recovery with enhanced fibrosis at 30 dpa in comparison to wild-type ventricles. Further, foxm1 was predominantly expressed in border zone cardiomyocytes and RNA-seq analysis of wild-type and foxm1m/m ventricles at 3 dpa uncovered decreased expression of cell cycle progression, insulin signaling, glycolysis, and AP-1 factors, implicating foxm1 as a regulatory factor in cardiomyocyte division. Moreover, this RNA-seq dataset identified diminished expression of cenpf in foxm1m/m ventricles in comparison to wild-type at 3 dpa. Examination of cenpf mutants demonstrated large fibrotic scars and increased cardiomyocyte binucleation at 30 dpa. Overall, the authors demonstrate the requirement of foxm1 and cenpf for effective cardiac regeneration; this will contribute to further findings and help isolate regulatory differences in zebrafish and mammalian injury responses in cardiomyocytes that could potentially be utilized to enhance regeneration in humans.

© 2023. Published by The Company of Biologists under the terms of the Creative Commons Attribution License (https://creativecommons.org/licenses/by/4.0/).

Reviewer 1 Comments for the Author: I am satisfied with the revised version and no more comments.

We thank the reviewer for taking the time to critically review this resubmission and appreciate their positive summary on this study.

Reviewer 2 Advance Summary and Potential Significance to Field: This manuscript is a reviewed version of a previous submission from Zuppo and colleagues. Overall, the authors have been only minimally responsive to my comments. Here are the specifics:

Reviewer 2 Comments for the Author: This manuscript is a reviewed version of a previous submission from Zuppo and colleagues. Overall, the authors have been only minimally responsive to my comments. Here are the specifics:

* The defect at 30 dpa in the foxm1 mutant remains rather unconvincing. The authors have not addressed my comment #2 regarding providing magnifications of the injured areas and complementing the AFOGs with some immunostainings to detect myocardial recovery - this foundational element of their paper should be much better supported. In 5 out of the 7 WT hearts shown in Fig. S2, I cannot see any of the stereotypical morphology of the regenerated myocardium after resection (expansion of the cortical myocardium, etc. - see Poss et al., 2002), so I'm not convinced the authors are comparing the same areas across genotypes.

* The methods used to quantify the scar tissue remain vague. How do the authors define the scar (see comment #2 in my previous review)? Blue pixels based on AFOG? Are they arbitrarily selecting an area manually? While measuring 60 sections per heart (PMID: 21473762) is excessive, measuring just four is insufficient.

We apologize for the oversight in not stating in the methods of our revised approach to quantify the scar tissue. For points 1 and 2, we have added new text in the methods section to state how scar tissue was quantified. The methods section now reads:

"….Hearts were embedded in Surgipath Cryo-Gel (Leica Biosystems) the following day, and samples were sectioned on a cryostat at 14 μm and were dried overnight at RT. For fibrosis staining, we performed Acid Fuchsin Orange G (AFOG) staining, which labels muscle as orange- brown, collagen as blue, and fibrin as red, as previously described (Poss et al., 2002). Images were taken with a Leica MZ16 microscope and Q Imaging Retiga 1300 camera. Fibrotic tissue was defined as the combined area of blue (collagen) and/or red (fibrin) pixels within the ventricular apex following apical resection. Fibrotic area was semi-qualitatively scored as 0 (no fibrotic tissue detected at the ventricle apex), 1 (small amount of fibrosis with some collagen and fibrin stain), or 2 (medium to large amount of fibrosis with collagen and fibrin stain). This method is preferrable to measuring the accumulation of collagen/fibrin in smaller areas due the variable between individual sections. Multiple sections were analyzed per individual heart. A quantitative calculation was measured as follows: fibrotic area and ventricle area were represented as pixels and were converted into microns squared (μm^2) by measuring the length (in pixels) of 1mm using the 5X objective. Scar area was calculated by dividing the fibrotic area by the ventricle area. The average scar area (μm^2) was determined using 4 sections per individual heart. All scoring and analysis were performed by an experimenter blinded to the individual animal's genotype."

* The authors need to incorporate some of the critical explanations the three reviewers have requested into their text. While I appreciate the responses in the "Answer to reviewers", they should include many of these pieces of information in the main text. If the reviewers asked for them, readers would likely have the same questions. For example, why are they not including the relevant information re- the difference in phenotypes between mammalian and zebrafish foxm1 mutants? (comment #1). The combined use of RNASeq from different genotypes (pointed out by reviewer 1) is also not justified in the text, just in the letter.

We agree with this comment and have now include explanations to all the points within the main text.

© 2023. Published by The Company of Biologists under the terms of the Creative Commons Attribution License (https://creativecommons.org/licenses/by/4.0/).

From reviewer 1 and 2 to address the combination of RNAseq from different genotypes we added this text:

"For analysis, WT and *dusp6* mutant data were combined based on their injury status as these were single bulk RNAseq experiments."

For reviewer 1's comment on difference in *foxm1* phenotype, we have included in the text the following:

"It was surprising that loss of *foxm1* does not result in an embryonic phenotype as has been reported in mice (Bolte et al., 2011; Sengupta et al., 2013). We did note that adult male *foxm1* homozygous mutants are infertile and have never been able to successfully fertilize eggs. The *foxm1* ENU-induced mutation from the Sanger Consortium is predicted to be within the forkhead DNA binding domain. Thus, we believe that the mutation is damaging, and it is not likely a splice variant can compensate for the forkhead DNA binding function. One reason for the lack of embryonic phenotype is that related Fox genes may compensate for the loss of *foxm1* during development."

In the discussion section on the Foxm1's role in fibrotic scar resolution.

"Beyond Foxm1 function in cardiomyocytes, other *foxm1* expressing cells could contribute to the fibrosis phenotype we observed. A recent study by Goda *et al.* demonstrated that macrophage-specific deletion of *Foxm1* increased pro-inflammatory cytokine expression and promoted pulmonary fibrosis(Goda et al., 2020). This suggests the macrophage profile in *foxm1*$^{m/m}$ hearts may have shifted from a pro-regenerative to pro-inflammatory phenotype which would impede the removal of fibrotic tissue."

In the Results section we include the following text to compare the differences in mouse vs zebrafish foxm1 mutants:

"We did observe that the male *foxm1* homozygous mutants are infertile and have never been able to successfully fertilize eggs suggesting that *foxm1* mutants do have a male fertility defect. The *foxm1* ENU-induced mutation from the Sanger Consortium is predicted to be within the forkhead DNA binding domain. Thus, we believe that the mutation is damaging, and it is not likely a splice variant can compensate for the forkhead DNA binding function. One reason for the lack of embryonic phenotype is that there exist many related Fox genes that have been documented to play a role in proliferation and may compensate for the loss of *foxm1*."

* The data presented in Sup Fig 6 re- the cenpf phenotype is, again, not convincing. In my opinion, the mutant heart looks regenerated. I cannot see the injured/regenerated area in the WT hearts.

* The lack of binucleation in foxm1 mutants is justified by the "lack of cell cycle activity", but this is an overstatement. Yes, there is significantly less cardiomyocyte proliferation (from around 20% to 10%), but if their hypothesis is true, there should be some increase in binucleation in these hearts.

For these two points we have now included representative images of the AFOG staining at 60dpa for WT and *cenpf* mutants (please see new Figure. S6). We have softened the language and changed the sentence. Specifically for the subtitle:

"cenpf mutants fail to regenerate due to increased cardiomyocyte binucleation"
to
"*cenpf* mutants exhibit increased cardiomyocyte binucleation"

The end sentence in paragraph:
"Therefore, the loss of *cenpf* caused mitotic dysregulation during heart regeneration, and because fewer cardiomyocytes divided, fibrotic scar tissue persisted."

© 2023. Published by The Company of Biologists under the terms of the Creative Commons Attribution License (https://creativecommons.org/licenses/by/4.0/).

To

Therefore, the loss of *cenpf* resulted in cardiomyocyte mitotic dysregulation during heart regeneration.

* I do not feel the authors have enough data to support the model from Fig. 6. As I read this drawing, they're proposing that loss of foxm1 results in regeneration failure involving G2/M checkpoint failure. If true, then cells would be 4n, but they have not demonstrated an increase in cardiomyocyte ploidy after injury.

We thank the reviewer for raising this issue about over interpretation of our data. To address this, we have included a paragraph discussing the limitations as suggested. The text in the discussion:

" In contrast, no increase of binucleated cardiomyocytes in *foxm1*$^{m/m}$ was detected, which we reason to be due to the lack of cell-cycle activity in the mutant hearts. This does not exclude the possibility of polyploidy as this phenomenon was reported in both cardiomyocytes and hepatocytes from *Foxm1* KO mice (Korver et al., 1998; Krupczak-Hollis et al., 2004; Ramakrishna et al., 2007) and it may be possible that *foxm1*$^{m/m}$ cardiomyocytes fail to progress beyond G2 and develop a 4n ploidy. Alternatively, since *FoxM1* mRNA and protein levels typically increase at the entry of S phase (Laoukili et al., 2008), it is also possible *foxm1*$^{m/m}$ cardiomyocytes fail to progress beyond the G1/S checkpoint and prematurely exit from the cell cycle without doubling their DNA content and remain as mononucleated diploid cells that exhibit a quiescent phenotype. Additional experiments will determine if premature cell cycle exit at the G1/S checkpoint or accumulation of 4n cardiomyocytes are reasons for the lack of proliferation in *foxm1* mutant hearts after cardiac injury."

In addition, we have changed the figure legend to include "a preliminary model" for Figure 6 and also modified the text to soften the language on our conclusions:

"**Figure 6: A Preliminary model indicating requirement of *foxm1* and *cenpf* expression in border zone cardiomyocyte cell cycle progression and mitosis.** In post-injury hearts, a sub- population of border zone cardiomyocytes express *foxm1*. Foxm1 transcriptionally activates expression of cell cycle genes such as *cenpf*, *g2e3*, and *prc1b* to promote regeneration of the lost myocardium. In the absence of *foxm1*, induction of these G2 and M phase genes are decreased, and border zone cardiomyocytes do not proliferate. In the absence of *cenpf*, cardiomyocytes may progress through the cell cycle until M phase when Cenpf protein is required for chromatid separation. These dividing cells likely undergo cytokinetic dysregulation which results in failed mitosis, generating binucleated cardiomyocytes."

We have also edited the Figure 6 to show that foxm1 mutants exhibit decreased G2 and M phase gene expression as what was previously showing that G2/M checkpoint failure. We agree this is misleading.

Reviewer 3 Advance Summary and Potential Significance to Field: This is a revised manuscript by Zuppo et al aims to identify the role of Foxm1 on zebrafish cardiomyocyte proliferation and regeneration. The authors have addressed my questions and I have no further comments.

Reviewer 3 Comments for the Author: The authors have addressed my questions and I have no further comments.

We thank the reviewer for taking time to read and review our resubmission. We thank them for their positive response.

© 2023. Published by The Company of Biologists under the terms of the Creative Commons Attribution License (https://creativecommons.org/licenses/by/4.0/).

Third decision letter

MS ID#: DEVELOP/2022/201163

MS TITLE: Foxm1 regulates cardiomyocyte proliferation in adult zebrafish after cardiac injury

AUTHORS: Daniel A Zuppo, Maria Azzurra Missinato, Lucas Santana-Santos, Guang Li, Panayiotis V Benos, and Michael Tsang
ARTICLE TYPE: Research Article

I am happy to tell you that your manuscript has been accepted for publication in Development, pending our standard ethics checks.

© 2023. Published by The Company of Biologists under the terms of the Creative Commons Attribution License (https://creativecommons.org/licenses/by/4.0/).
